# Host-derived growth factors drive ERK phosphorylation and MCL1 expression to promote osteosarcoma cell survival during metastatic lung colonization

**DOI:** 10.1007/s13402-023-00867-w

**Published:** 2023-09-07

**Authors:** Camille A. McAloney, Rawan Makkawi, Yogesh Budhathoki, Matthew V. Cannon, Emily M. Franz, Amy C. Gross, Maren Cam, Tatyana A. Vetter, Rebekka Duhen, Alexander E. Davies, Ryan D. Roberts

**Affiliations:** 1 Department of Veterinary Biosciences, College of Veterinary Medicine, The Ohio State University, Columbus, OH USA; 2 Center for Childhood Cancers and Blood Diseases, Abigail Wexner Research Institute at Nationwide Children’s Hospital, Columbus, OH USA; 3 Knight Cancer Institute’s, Cancer Early Detection Advanced Research Center, Oregon Health & Science University, 3181 SW Sam Jackson Park Road, Portland, OR 97239 USA; 4 Molecular, Cellular, and Developmental Biology Program, The Ohio State University, Columbus, OH USA; 5 Center for Gene Therapy, Abigail Wexner Research Institute, Nationwide Children’s Hospital, Columbus, OH USA; 6 Division of Pediatric Hematology, Oncology, and BMT, Nationwide Children’s Hospital, 700 Children’s Drive, Columbus, OH 43205 USA; 7 The Ohio State University James Comprehensive Cancer Center, Columbus, OH USA; 8 Department of Pediatrics, The Ohio State University, Columbus, OH USA

**Keywords:** Osteosarcoma, Metastasis, MCL1, MAPK, pERK

## Abstract

**Purpose:**

For patients with osteosarcoma, disease-related mortality most often results from lung metastasis—a phenomenon shared with many solid tumors. While established metastatic lesions behave aggressively, very few of the tumor cells that reach the lung will survive. By identifying mechanisms that facilitate survival of disseminated tumor cells, we can develop therapeutic strategies that prevent and treat metastasis.

**Methods:**

We analyzed single cell RNA-sequencing (scRNAseq) data from murine metastasis-bearing lungs to interrogate changes in both host and tumor cells during colonization. We used these data to elucidate pathways that become activated in cells that survive dissemination and identify candidate host-derived signals that drive activation. We validated these findings through live cell reporter systems, immunocytochemistry, and fluorescent immunohistochemistry. We then validated the functional relevance of key candidates using pharmacologic inhibition in models of metastatic osteosarcoma.

**Results:**

Expression patterns suggest that the MAPK pathway is significantly elevated in early and established metastases. MAPK activity correlates with expression of anti-apoptotic genes, especially MCL1. Niche cells produce growth factors that increase ERK phosphorylation and MCL1 expression in tumor cells. Both early and established metastases are vulnerable to MCL1 inhibition, but not MEK inhibition in vivo. Combining MCL1 inhibition with chemotherapy both prevented colonization and eliminated established metastases in murine models of osteosarcoma.

**Conclusion:**

Niche-derived growth factors drive MAPK activity and MCL1 expression in osteosarcoma, promoting metastatic colonization. Although later metastases produce less MCL1, they remain dependent on it. MCL1 is a promising target for clinical trials in both human and canine patients.

**Supplementary Information:**

The online version contains supplementary material available at 10.1007/s13402-023-00867-w.

## Introduction

Survival in osteosarcoma, the most common primary tumor of bone, hinges almost entirely on the presence or absence of metastasis. More than 80% of osteosarcoma patients that develop metastasis die within five years, compared to 30% of patients with localized disease [1–3]. Metastasis often occurs months to years after detectable disease has been eliminated by surgery and chemotherapy. The vast majority of osteosarcoma metastases occur in the lungs. However, despite this striking tropism, it is thought that the lung is initially a hostile environment for tumor cells [4–8]. Indeed, tumor cells must overcome a number of obstacles to colonize the lungs including escaping the primary tumor, surviving anoikis and shear stress, evading the host’s immune system, and ultimately entering and proliferating in the lung. This “metastatic bottleneck” results in attrition of the majority of tumor cells, with the narrowest point of the bottleneck being the early survival of tumor cells upon dissemination [9–11]. Thus, understanding the mechanisms that determine survival in the early/establishing metastatic niche is imperative: disrupting these mechanisms could reduce or prevent metastasis, saving the lives of patients who have seen no effective improvement in outcome for several decades [1, 2.

Numerous studies have suggested that the mitogen-activated protein kinases (MAPK) pathway (also known as the RAS-RAF-MEK-ERK pathway) plays a role in osteosarcoma metastasis, however the functional consequences and therapeutic implications of its activation have not be elucidated [12–20]. The MAPK pathway is a highly conserved signaling cascade that is canonically activated by the binding of growth factors to tyrosine kinase receptors, many of which are found in osteosarcoma patient samples and cell lines [13, 17, 21–27]. ERK has been broadly implicated in a number of pro-tumorigenic functions, including transcription and stabilization of anti-apoptotic proteins MCL1, BCL2, and BCLXL [12–15, 18–20, 28–32].

As a member of the BCL2 family, MCL1 is known primarily for its role in inhibiting apoptosis through its interactions with anti-apoptotic proteins BAX and BAK [33]. Numerous tumor types have been shown to utilize increased MCL1 expression to promote survival, including multiple myeloma, small cell lung cancer, and others, suggesting MCL1 could also confer a survival advantage to metastatic osteosarcoma cells in the hostile lung environment [34–36]. Indeed, recent studies have shown that some osteosarcoma cell lines express more MCL1 than other BCL2 members [37, 38]. However, mechanisms of this increased expression have not yet been investigated, nor have studies determined what, if any, role this expression may play in tumor metastasis.

Our data provide insights into how osteosarcoma metastases survive in the metastatic niche and how tumor-host interactions may drive this survival. We found that niche cells produce growth factors that increase ERK activity and MCL1 expression in tumor cells, and that these growth factors are transcribed more highly in metastasis-bearing lungs. Although early metastases were shown to express the most MCL1 protein, we found that both early and later, established metastatic lesions were highly susceptible to MCL1 inhibition in a murine model of metastasis, confirming MCL1’s importance in metastatic tumor cell survival. Co-treatment with cyclophosphamide further improved the efficacy of the MCL1-specific BH3 mimetic AZD5991, reducing and even eliminating detectable metastatic disease in mice. Targeting MCL1 in osteosarcoma shows therapeutic potential and may be a viable strategy for canine and human clinical trials, as dogs are an established model of spontaneous osteosarcoma and their MCL1 protein is highly homologous to humans [39–49]. Screening therapeutics in complex, immunocompetent models–such as dogs–may help ensure drugs with a higher chance of success are used in human clinical trials [9, 48, 50].

## Methods

### Experimental models – patient derived xenografts (PDXs), cell lines, and murine studies

*PDXs*. OS-17 PDX tissue was obtained from a primary femur biopsy performed at St. Jude’s Children’s Research Hospital in Memphis and was a gift from Peter Houghton [51].

*Cell lines*. OS-17 cells were derived from an early passage of the OS-17 PDX (see above). These cells were grown in RPMI (Corning, 10-040-CV) supplemented with 10% fetal bovine serum (FBS) (R&D Systems, S11150H). Metastatic OS-17 tumors collected 2 weeks after injection were considered “early”, those collected 3–4 weeks considered “midpoint”, and those collected 5–7 weeks considered “late”.

MG63.3 cells were a gift from Dr. Poul Sorensen under a materials transfer agreement (MTA) with NCI (Franklin) and grown in DMEM (Corning, 10-013-CV) supplemented with 10% FBS. Metastatic MG63.3 tumors collected 1 week after injection were considered “early,” those collected 2–3 weeks were considered “midpoint”, and those collected 4–5 weeks considered “late”.

HBEC3-KT are hTERT-immortalized human bronchial epithelial cells obtained from the American Type Culture Collection (ATCC CRL-4051) and grown in Airway Epithelial Cell Basal Medium (ATCC PCS-300-030) supplemented with the contents of the Bronchial Epithelial Growth Cell Kit (ATCC PCS-300-030).

Primary, Normal, Human Lung Fibroblasts (HLF) were obtained from ATCC (PCS-201-013) and grown in fibroblast basal medium (PCS-201-030) supplemented with fibroblast growth kit-low serum (PCS-201-041).

143B cells were obtained from ATCC (CRL-8303) and grown in DMEM (Gibco, 11,965,092) supplemented with 10% FBS. All cell lines were utilized within 20 passages of the original aliquots. All cell lines were tested annually for authenticity using short tandem repeat (STR) genotyping and mycoplasma contamination using commercial services provided by Lab Corporation of America.

*Isolation of human monocytes*. Whole blood samples were collected in EDTA-anticoagulated vacutainers from adult donors with no previous history of cancer. Within two hours of collection, blood samples were processed according to a PBMC isolation protocol using density-gradient centrifugation with Ficoll (Cytiva, 45-001-752). RPMI media was used for blood dilution and pellet resuspension. EasySep™ Human Monocyte Isolation Kit (Stemcell Technologies, 19,359) was then used to isolate monocytes from the PMBCs.

*Generation of OS-17-FLUC cells*. Pre-made lentivirus carrying Firefly luciferase (Cellomics Technology, PLV-10064-50) was used to infect OS-17 cells in the presence of Polybrene (8 µg/ml) (MilliporeSigma, TR1003G).

*Generation of niche labeling OS-17 cells (slp-mCherry OS-17) in experimental metastases.* Lentivirus carrying pcPPT-mPGK-attR-sLPmCherry-WPRE (gift from Ilaria Malanchi, Ximbio #155,083) was synthesized using pCMV-VSV-G (gift from Bob Weinberg [Addgene plasmid # 8454; http://n2t.net/addgene:8454; RRID:Addgene_8454]) and pCMV delta R8.2 (gift from Didier Trono [Addgene plasmid # 12,263; http://n2t.net/addgene:12263; RRID:Addgene_12263]) as previously described​ with polyethylenimine (PEI), (Alfa Aesar, 43,896) as the transfection reagent [52–55]. OS-17 cells were infected with this virus as described above. Three days after infection, slp-mCherry OS-17 cells were isolated using a BD Influx cell sorter (BD Biosciences, 23-10536-00) using 610/20 filter to > 90% purity. Cells were maintained on media supplemented with 1x anti-anti (Gibco, 15240-062) for 1 week after sorting and tested for mycoplasma and STR prior to use as described above.

*Generation of reporter* cells *(OS-17 ERKTR mVenus H2B iRFP670)*. Lentivirus carrying pLJM1-ERKTR-mVenus-puro (gift from John Albeck) was produced using psPAX2 (gift from Didier Trono [Addgene plasmid # 12,260; http://n2t.net/addgene:12260; RRID:Addgene_12260]) and pMD2.G (gift from Didier Trono [Addgene plasmid # 12,259; http://n2t.net/addgene:12259; RRID:Addgene_12259]) as previously described​ with PEI as the transfection reagent. Lentivirus carrying pLJM1-H2B-iRFP670-puro (gift from Heather Shive) was also produced using the same packaging plasmids and PEI. Cells were transduced with both viruses in the presence of polybrene, then selected for reporter integration using 1 µg/mL puromycin. After selection, cells were flow sorted using a BD Influx cell sorter using 530/40 and 720/40 filters, and double positive cells were collected with > 90% purity. Cells were maintained on media supplemented with 1x anti-anti for 1 week after sorting and tested for mycoplasma and STR prior to use as described above.

*Murine Studies*. All animal studies were approved by Nationwide Children’s Hospital Institutional Animal Care and Use Committee (IACUC protocol AR15-00022).

*Experimental metastasis timepoints and primary tumors.* Single cell suspensions of 1 × 10^6^ OS-17, OS-17-FLUC or slp-mCherry OS-17 cells were injected intra-venously in six to eight week old female C.B-17/IcrHsd-Prkdc^scid^ mice. Mice were humanely euthanized 2, 4, 6, and 7 weeks after injection. Control mice, which had not been injected with tumor cells, were also humanely euthanized. Single cell suspensions of 5 × 10^5^ OS-17 cells were injected intratibially in C.B-17/IcrHsd-Prkdc^scid^ mice (Envigo, Frederick, MD). Mice were humanely euthanized once tumors reached 1000 mm^3^.

Single cell suspensions of 1 × 10^6^ MG63.3 cells were injected intra-venously in six to eight week old female C.B-17/IcrHsd-Prkdc^scid^. Mice were humanely euthanized at 1, 3, and 5 weeks after injection. Single cell suspensions of 5 × 10^5^ MG63.3 cells were injected intratibially in C.B-17/IcrHsd-Prkdc^scid^ mice. Mice were euthanized once tumors reached 1000 mm^3^.

In all cases, after euthanasia lung and tumor tissue was processed according to our scRNAseq protocol, fluorescent immunohistochemistry (IHC-F) protocol, and/or Western blot protocol, below.

#### Drug efficacy testing in experimental metastasis

Single cell suspensions of 1 × 10^6^ cells of OS-17 or OS-17-FLUC were injected intra-venously (IV) in C.B-17/IcrHsd-Prkdc^scid^ mice. AZD5991 (MedChemExpress, HY-101,533) was dissolved in 10% dimethyl sulfoxide (DMSO) (Fisher BioReagents, BP231-100), 40% polyethylene glycol (PEG)-300 (Fluka Analytica, 25322-68-3), and 5% Tween-80 (Acros Organics, 9005-65-6) in sterile saline (0.9% NaCl). Mice were treated with 60 mg/kg AZD5991 intraperitoneally (IP) for all doses on a Monday-Wednesday-Friday schedule for six doses unless otherwise noted (Supplemental Fig. 1). Cyclophosphamide (MedChemExpress, HY-17,420 A/CS-5005) was dissolved in 5% DMSO, 30% PEG-300, and 5% Tween-20 (Fisher Scientific, BP337-500) in sterile saline. Mice were treated with 100 mg/kg cyclophosphamide IP once per week for two weeks. Trametinib (MedChemExpress, HY-10,999) was dissolved in 10% cremophor EL (Calbiochem, 238470-1SET), 10% PEG-300, and sterile saline. Mice were treated with 1 mg/kg or 0.1 mg/kg, depending on the treatment group, per oral (PO) once a day for 27 days. For the pilot study, three mice were assigned per group and treatments began 24 h after inoculation with tumor cells. Mice in the AZD5991 groups and AZD5991 vehicle group all received the first dose of AZD5991 or vehicle IV, and subsequent doses were given IP.

For the study testing efficacy of AZD5991 and cyclophosphamide alone and in combination, forty-five mice were injected with single cell suspensions of 1 × 10^6^ cells of OS-17-FLUC intra-venously and treatments began on day 28. After three weeks, mice were anesthetized with isoflurane and imaged using the IVIS Spectrum In Vivo Imaging System (PerkinElmer, 124,262) following IP injection with 150 mg/kg luciferin (Gold Bio, LUCK-1G). The resulting luminescence minus background (measured in radiance [photons]) was evaluated in Living Image (Version 4.7.4; PerkinElmer; Hopkinton, MA, USA) and used to randomize mice into groups to equalize tumor burden). After imaging mice were monitored to ensure they recovered appropriately. Each treatment group had ten mice for a total of forty; the five mice with the lowest luminescence were removed from the study. Mice were imaged again prior to treatment, and weekly throughout the course of treatment until endpoint.

At study end point, or if a mouse met endpoint criteria, mice were humanely euthanized via CO_2_ inhalation followed by sternotomy. Endpoint criteria for euthanasia was defined as weight loss of ≥ 20% or an enhanced body condition score (eBCS) of ≤ 9 [56] Following humane euthanasia, lungs were flushed via slow instillation of phosphate-buffered saline (PBS) (Corning, 21-031-CV) into the right ventricle until lungs were cleared of blood. Lungs were dissected from each mouse *en bloc* along with trachea and heart. Lungs were slowly instilled with 10% neutral buffered formalin (NBF) until fully expanded, then the trachea was tied off with suture andplaced in NBF for 24 h at 4 °C. For hematoxylin and eosin (H&E) staining and IHC-F studies, tissues were then moved to PBS for at least one hour at 4 °C. The right lobes of each lung were then processed for formaldehyde fixed paraffin embedding (FFPE) via standard techniques. The left and accessory lobes of the lungs per mouse were placed in a 30% sucrose PBS solution for 24 h at 4 °C prior to embedding in optimal cutting temperature media (OCT) (Fisher HealthCare, 23-730-571) for 10 min at room temperature. Sections were then placed on dry ice until fully opaque, then wrapped in foil and stored at − 80 °C.

### Live cell imaging

Imaging was performed on 96-well plates with #1.5 glass bottoms (Cellvis, P96-1.5 H-N) coated using 5 µl of 50 µg/mL rat tail collagen Type I (ThermoFisher, A1048301) dissolved in 20 mM acetic acid for one hour then washed with PBS to remove excess collagen. OS-17 reporter cells were then seeded at a density of 10,000 cells per well and left for one hour to adhere. Cells were left to incubate overnight in RPMI (Gibco, 11875-119) with 10% FBS (GeminiBio, 900 − 108). After incubation, cells were washed twice with phenol-free DMEM/F-12 (Gibco, 21041-025) with no additives, then placed in imaging media consisting of phenol-free DMEM/F-12 containing 1.4 × 10^–6^ M hydrocortisone, 10^–10^ M β-estrogen, and 10 µg/ml transferrin, and left to incubate for 4 h before imaging. Live-cell imaging was performed on a Nikon Ti2 automated microscope fitted with an Oko-Lab environmental chamber maintained at 37 °C and 5% CO_2_, SOLA II LED illumination source, Photometrics Prime 95B or Orca Fusion BT camera, and controlled with NIS-Elements AR software. Images of each well were collected every 6 min for the duration of the experiment. Identical imaging settings were used for all samples within the same imaging set. Each image was captured with a Nikon Plan Apo 0.80 NA 20X air objective. Cells were initially imaged for four hours to obtain baseline ERK phosphorylation. After four hours, the following recombinant human growth factors were added to three wells each at a concentration ranging from 1 to 100 ng/mL: PDGF-CC (PeproTech, 100-00CC), PDGF-BB (PeproTech, 100-14B), PDGF-AA (PeproTech, 100-13 A), TGF-β (R&D Systems, 7754-BH), TGF-α (PeproTech, 100-16 A), FGF18 (PeproTech, 100 − 28), FGF10 (PeproTech, 100 − 26), FGF7 (PeproTech, 100 − 19), HBEGF (PeproTech, 100 − 47), NRG1 (PeproTech, 100-03), EREG (PeproTech, 100-04), AREG (PeproTech, 100-55B), EGF (PeproTech, AF-100-15), FGF2 (PeproTech, 100-18B), IGF1 (PeproTech, AF-100-11), HGF (PeproTech,100-39 H) and VEGF-A (PeproTech,100-20B). Control wells were treated with imaging media (described above). Cells were imaged for an additional 15 h, after which 100 nM of the MEK inhibitor (MEKi) PD0325901 (Selleckchem, S1036) was added to obtain minimum ERK activity.

### Live cell image processing and analysis

Images were analyzed using MATLAB R2022b (Mathworks, Natick, MA, USA). Custom MATLAB processing code (provided by the Micheal, Pargett, Albeck Lab, UC Davis) was applied to live-cell imaging data to segment and track individual cells via the histone H2B nuclear fluorescence, and to obtain ERKTR nuclear and cytoplasmic fluorescence intensities to quantitatively assess reporter activity as previously described [57, 58] Processed data from three independent replicate experiments, with a minimum of 500 cells per condition, were baseline normalized to MEK-inhibited ERKTR and combined for all reported analyses herein. Growth factor responder analysis was performed in MATLAB, whereby a paired t-test was used to determine statistically significant changes in single cell ERKTR signals pre- versus post-treatment with growth factor or inhibitor (significance was considered at P < 0.005) [59] Individual cells exhibiting a significant response were further analyzed to obtain single cell maximum response amplitude within 1 h of growth factor addition and area under the curve for 12 h was assessed as surrogate for duration of response using MATLAB software.

### Metastatic organoids and live-cell imaging co-cultures

To form metastatic organoids, 0.4 µM inserts were placed into 24 well plates. A total of 25,000 HBEC3-KT cells were added to each insert in 200 µL of Airway Epithelial Cell Basal Medium supplemented with the contents of the Bronchial Epithelial Growth Cell Kit. The well below was filled with 700 µL of the same media. Cells were left to grow until fully confluent, 24 to 72 h, at which point the media within the insert was aspirated and replaced with serum-free DMEM containing 15,000 OS-17 pERK reporter cells per insert. Media below the insert in the well was aspirated and replaced with 700 µL of DMEM with 1% FBS and 1x insulin transferrin selenium (Gibco, 41,400,045). The next day, inserts were examined for the presence of metastatic organoids. Each insert was photographed twice with a 10x objective using light microscopy and GFP fluorescence to differentiate tumor cells from HBEC3-KT cells. After photographing, the media below the insert in the well was replaced with fresh DMEM with 1% FBS and 1x insulin transferrin selenium. Trametinib (dissolved in DMSO) or AZD5991 (dissolved in DMSO) were added to the media of each well as necessary to achieve the desired concentration; DMSO (1:1000 concentration) was added to each control well. 24 h later each insert was photographed again, followed by an identical media change. This was repeated for a total of 72 h. Metastatic organoids in each well were counted using Adobe Photoshop’s counting tool (Version 23.5.1; Adobe, Inc.; San Jose, CA, USA), with the counts from both photos per well combined to give the total number of spheroids per well. The percentage change of metastatic organoids was calculated by dividing the number of organoids present at the end of the experiment by the number of pre-treated organoids. Data were subjected to one-way analysis of variance (ANOVA) of multiple comparisons of experimental condition group means against the mean of a control group. Multiple comparisons were corrected for by controlling the False Discovery Rate using the original method of Benjamini and Hochberg.

HLF cells were seeded onto collagen coated 96-well plates with #1.5 glass bottoms and cultured until confluent. Monocytes and OS-17 reporter cells were seeded at a density ratio of 3:1 on top of the confluent layer of HLF cells. Monoculture of OS-17 cells was seeded directly onto collagen coated plate at a similar cell density as OS-17 cells in co-culture conditions. The monoculture and co-culture were covered in a thin layer of 5% Cultrex Reduced Growth Factor Basement Membrane Extract (R&D Systems, 3536-005-02). The co-culture was incubated in RPMI media supplemented with 10% FBS for a period of 72 h. Media was then changed to imaging media and cells were left to incubate for 72 more hours. Cells were then fixed, stained, and imaged as described under Immunocy­tochemistry.

### Single-cell RNA-sequencing (scRNAseq) tissue processing

Tumors harvested from mice were processed using the human tumor dissociation kit (Miltenyi Biotec, 130-095-929) with a GentleMacs Octo Dissociator with Heaters (Miltenyi Biotec, 130-096-427). Lungs from a single mouse were used for the control timepoint, lungs from two mice were pooled for the 2 week timepoint, lungs from two mice were pooled for the 4 week timepoint, lungs from one mouse were used for the 7 week timepoint, and the primary tumor from one mouse was used for the primary timepoint. OS-17 and niche cells expressing slp-mCherry were isolated using a BD Influx cell sorter using laser 610/20 BP. Single cell suspensions in 0.04% bovine serum albumin (BSA) (R&D Systems, S11150) -PBS of dissociated tumor tissues were generated and run on Chromium Single Cell 3′RNA-sequencing system (10X Genomics) with the Reagent Kit v3.1 (10X Genomics, PN-1,000,121) according to the manufacturer’s instructions. Briefly, cells were loaded into Chromium Next GEM Chip G Single Cell Kit (10X Genomics, PN-1,000,120) with a targeted cell recovery of 5,000–8,000 cells per sample. After performing cDNA purification, amplification, and library construction (sample index PCR cycles determined by protocol) as instructed, we sequenced sample libraries on a half lane of HS4000 (Illumina), SP, or NS_S2 to yield (after quality control) about 65,000 paired-end reads per cell.

### scRNAseq analysis

We used Cell Ranger (v3.0.2, 10X genomics) to align the reads to a joint human/mouse (hg19/mm10) genomic reference and generate gene count data per cell [60]. We analyzed the output datasets in R using the Seurat package [61–64]. We filtered out cell fragments and droplets with multiple cells using sample specific minimum and maximum allowed values for the number of unique molecular identifiers (UMIs) per cell. We also filtered out dead or dying cells using a maximum value cutoff for the percentage of mitochondrial reads per cell. We then merged the datasets and used Seurat to normalize and scale the data for downstream analysis. We retained 9,580 tumor and 19,979 mouse stromal cells with 9,682 unique UMIs per cell on average. Mouse stromal cell types were annotated by comparing assignments made by the SingleR R package using the ImmGenData and MouseRNAseqData references from the celldex R package, as well as a normal lung reference derived from publicly available data [65, 66]. We also looked at expression of cell type markers outlined in several publications to assess cell type assignment accuracy [67–70]. For final cell type assignments, we clustered the data at high resolution and assigned each cluster to a consensus cell type. We performed differential expression analysis on the single cell data using the FindMarkers function within the Seurat package, using the default statistical test (Wilcoxon rank sum test) and a logfc.threshold setting of 0 to keep results for all genes.

To explore biological pathways differentially enriched between cell types and timepoints, we used the fgsea R package to perform geneset enrichment analysis (GSEA) with the pathways retrieved using the msigdbr R package [71]. We evaluated upstream regulators of differential expression analysis results using the NicheNet R package [72, 73]. We also used the Ingenuity Pathways Analysis (IPA) software to perform pathway analysis on differential expression analysis results [74].

All code used to generate these analyses are freely available on GitHub (https://github.com/kidcancerlab/MCL1_early_met_survival).

### H&E staining

Paraffin embedded tissues were cut into 4 µM sections and placed on glass slides. The slides were heat fixed at 60 °C for at least thirty minutes, then dried at 60 °C in an oven for 20–60 min. The sections were deparaffinized with xylene (Fisher Scientific, X3P-1Gal) and rehydrated through ethanol series (Fisher Scientific, 04-355-223). Slides were submerged in hematoxylin (Sakura, 6190) for four minutes, then rinsed with running tap water for two minutes. Slides were submerged in acid alcohol (Sakura, 6190) for 10 s, then rinsed with running tap water for two minutes. Slides were submerged in bluing reagent (Sakura, 6190) for 10–30 s, followed by a rinse with running tap water for two minutes. Slides were submerged in 95% ethanol for 30 s and then Eosin-Y (Sakura, 6190) for two minutes, followed by 95% ethanol for 30 s. Slides were then submerged in 100% ethanol for one minute, twice. Slides were submerged in xylene for 2 min, twice. A coverslip was then placed on slides using Permount (Fisher Scientific, SP15-500).

### Fluorescent immunohistochemistry

Primary antibodies against human MCL1 (Cell Signaling Technology, D5V5L, 1:50), multi-species MCL1 (Abcam, ab28147, 1:100), multi-species Fra-1 (Santa Cruz Biotechnology, sc-28,310, 1:50), human vimentin (Abnova, SRL33, 1:200), and multi-species vimentin (Cell Signaling Technology, 5741 S, 1:00 or R&D, MAB2105, 1:100) were used. Secondary antibodies and counterstain used were donkey anti-rabbit Alexa Fluor 488 antibody (Invitrogen, A21202, 1:500), goat anti-mouse Alexa Fluor Plus 488 antibody (Invitrogen, A32723, 1:500), donkey anti-mouse Alexa Fluor 568 antibody (Invitrogen, A10037, 1:500), donkey anti-rabbit Alexa Fluor 568 antibody (Invitrogen, A10042, 1:500), donkey anti-rat Alexa Fluor 568 antibody (Invitrogen, A78946, 1:500), and 4, 6-diamidino-2-phenylindole dihydrochloride (DAPI) (Invitrogen, D1306, 1:500).

Paraffin embedded tissues were cut into 4 µM sections and placed on glass slides. The sections were deparaffinized with xylene and rehydrated through ethanol series. Sections were submerged into a citrate solution (pH 6.0) and heated for antigen retrieval. OCT embedded tissues were cut into 10 µM sections and mounted on Bond380 slides (Microscopy Sciences, 63,700-B1). These OCT embedded sections were brought to room temperature and then rehydrated in PBS for ten minutes. Both paraffin embedded and OCT embedded sections were blocked and permeabilized with a solution of PBS + 0.2% Triton x-100 (Sigma-Aldrich, 10,789,704,001) (v/v) + 2% BSA (w/v) for one hour at room temperature. Primary antibodies were diluted in this same solution. The blocking solution was removed from slides and primary antibodies were applied to the sections overnight at 4 °C. Sections were washed three times with PBS + 0.2% Triton x-100. All secondary antibodies and DAPI were diluted in PBS + 0.2% Triton x-100 + 2% BSA and added to the samples for 1 h at room temperature. Sections were washed three times with PBS + 0.2% Triton x-100, and once a final time with reverse osmosis water. Samples were mounted with Fluoromount-G (Invitrogen, 00-4958-02). Each tissue section was co-stained for vimentin and one of the markers of interest and counter-stained with DAPI. For secondary only controls, tissue sections were stained with only a vimentin primary antibody, DAPI, and anti-rabbit and anti-mouse secondaries. When quantifying tumor number, as well as lung tissue and tumor area for evaluating metastatic burden, only vimentin was stained for, with DAPI as a counterstain.

#### Evaluating MCL1 and Fra-1 expression in tissue

Multichannel images were captured using a Zeiss LSM 800 confocal microscope and ZEN (version 3.7.97.03000; Carl Zeiss Microscopy GmbH; Jena, Germany). Ten individual tumors were imaged per time point using a Zeiss Plan Apochromat 63x oil immersion objective and the 405, 488, and 561 nm laser lines at a resolution of 0.1 μm/pixel in 16 bits. One image per time point of secondary only control was imaged as well. Identical imaging settings were used for the protein of interest for all samples within the same staining and imaging set. The secondary only control at each time point was used to adjust the lookup table (LUT) for each corresponding MCL1-stained time point to ensure background was removed from images.

#### Evaluating patient tissue for MCL1 expression

Tissue from human patient osteosarcoma metastases were obtained from patients consented under a protocol approved by the Nationwide Children’s Hospital Institutional Review Board. Tissue from canine patient osteosarcoma metastases were obtained from the Biospecimen Repository at The Ohio State University Veterinary Medical Center Blue Buffalo Veterinary Clinical Trials Office. All samples were formalin fixed, paraffin embedded and processed and stained for MCL1 and vimentin as described above. Multichannel images were captured using a Zeiss LSM 800 confocal microscope and ZEN (version 3.7.97.03000; Carl Zeiss Microscopy GmbH; Jena, Germany). Images were captured using a Zeiss Plan Apochromat 63x oil immersion objective and the 405, 488, and 561 nm laser lines at a resolution of 0.1 μm/pixel in 16 bits. For each image, LUTs were adjusted individually to eliminate background and optimize visualization.

#### Quantifying tissue fluorescence

Ten captured images per timepoint were analyzed using Nikon NIS-Elements AR software version 5.30 with the General Analysis 3 module for both Fra-1 and MCL1. A mask was set to localize on the channel of vimentin staining (tumor) staining. The 90th percentile intensity of the channel of Fra-1 or MCL1 staining was recorded within this mask.

#### Evaluating tumor number and metastatic burden

Whole-section multichannel images were captured of the entire tissue slices using a Nikon Eclipse Ti2-E motorized microscope with a Nikon D-LEDI multiline light engine, a Hamamatsu ORCA Fusion camera, and Nikon 20x Plan Apochromat Lambda D objective using Nikon NIS-Elements AR version 5.41 software. A DAPI/FITC/TRITC/CY5 quad cube and emission cleanup filters from Semrock were used to achieve complete signal separation. Each sample was scanned at 20x magnification with a final 16-bit image resolution of 0.32 μm/pixel. Identical imaging settings were used for all samples within the same staining and imaging set. Both stained and secondary only control tissues were scanned. All images were compressed by 20% to enable faster processing. Images were analyzed using Nikon NIS-Elements AR software version 5.30 with the General Analysis 3 module. All images were compressed by 50% to enable faster processing speed prior to analysis. In the first analysis algorithm, automated tissue segmentation was performed using an auto-threshold for DAPI signal, and tumor segmentation was performed using an auto-threshold for vimentin signal. Segmented regions were then manually modified by an unbiased operator as needed to ensure specific and complete selection of tumor and tissue. Images with both binary layers were then processed through a second algorithm where the total lung area, tumor area, and tumor count were measured. Metastatic burden was calculated by dividing the lung area by the area of all metastases and multiplying by 100. To ensure this method of quantifying tumor number and metastatic burden was accurate and precise, one hematoxylin and eosin-stained section per treatment group was given to two unbiased operators for a total of eight sections. These operators manually quantified tumor number, as well as tumor area and lung area to calculate metastatic burden. The results of hematoxylin and eosin-stained, fully manually evaluated sections and vimentin- and DAPI-stained semi-automated sections were then compared and showed a limit of agreement of -26 to 46 (tumor count) and − 1.52 to 1.45 (metastatic burden) when evaluated using Bland-Altman plot analysis. After semi-automated tumor segmentation was validated, the number of metastases and metastatic burden were determined using this method as described above. Data were then subjected to one-way ANOVA with post-hoc multiple comparisons relative to the combination treatment group.

### Immunocytochemistry

An antibody against human MCL1 (Cell Signaling Technology, D5V5L, 1:200) and secondary antibody goat anti-rabbit Alexa Fluor 546 (Invitrogen, A-11,035, 1:400) were used to evaluate MCL1 staining. An antibody against human Fra-1 (the protein product of the gene *FOSL1*) (Santa Cruz Biotechnology, sc-28,310, 1:400) and secondary antibody donkey anti-mouse Alexa Fluor 488 (Invitrogen, A21202, 1:400) were used to evaluate Fra-1 staining. Wild Type OS-17 or 143B cells were plated in glass bottom 96 well plates as described for live-cell preparations and incubated overnight in RPMI supplemented with 10% FBS (for OS-17) or DMEM supplemented with 10% FBS (for 143B). Cells were washed with phenol-free DMEM/F-12 with no additives, then placed in imaging media. Cells were then treated with PDGF-BB, TGF-a, FGF2, FGF18, HBEGF, HGF, IGF-1, EGF, ERG, and AREG at a concentration of 100 µg/ml for 24 h. The drug PD0325901 (100 nM) was used for MEKi treatments.

After 24 h, the media was aspirated from wells and replaced with 4% paraformaldehyde (Sigma-Aldrich, 47608-250ML-F) at room temperature for 20 min. The cells were washed once with PBS. Lipid was extracted from cells using PBS + 1% Triton X-100 for 10 min at room temperature. Cells were washed with PBS twice. Cells were blocked with blocking buffer (PBS containing 0.1% Triton X-100 and 2% BSA) for one hour at room temperature. Primary antibody was diluted in blocking buffer and left to incubate overnight at 4 °C. Cells were washed three times with PBS containing 0.1% Triton X-100, five minutes per wash at room temperature. The secondary antibody was diluted in blocking buffer and left to incubate for one hour at room temperature in the dark. Cells were washed three times with PBS containing 0.1% Triton X-100, five minutes per wash at room temperature. PBS was added to the wells to maintain hydration during imaging. Nuclei were stained with Hoechst-33,342 (1:10,000).

A single multichannel image of each well was captured using the Nikon Ti2 automated microscope previously described for live cell imaging. Each sample was scanned at 20x magnification. Identical imaging settings were used for all samples within the same staining.

Processing of fixed immunocytochemistry images was performed using the same MATLAB processing code used for live cell images to segment and extract nuclear and cytoplasmic fluorescence intensities. MCL1 signal was obtained by measuring the cytoplasmic signal based on its localization pattern, whereas Fra-1 was assessed in the nuclear channel. Signals were normalized to control condition. Statistical analysis for immunocytochemistry was done using MATLAB software and significance was considered at P < 0.05 for all statistical analyses. Two-tailed t-test was conducted to establish significance.

### Western blot

Tumors and control tissue harvested from mice were immediately snap frozen by complete submersion in liquid nitrogen. Snap frozen tissue was then powdered using instruments cooled with liquid nitrogen. Total protein was extracted using RIPA buffer (Thermo Scientific, 89,901) containing protease and phosphatase inhibitors (Thermo Scientific, 78,430 and Active Motif, 105,840). and quantified using a bicinchoninic acid (BCA) kit (Thermo Scientific, 23,227. Equal amounts of protein lysate were run on an SDS-PAGE gel (Invitrogen, NW04122BOX). and transferred onto a PVDF membrane (Invitrogen, LC2002). Membrane was blocked using a 3% BSA in tris-buffered saline and Tween-20 (TBST) solution (Epredia, TA-999-TT) for 1 h at room temperature, followed by incubation with primary antibodies overnight at 4 °C. Membranes were then washed with TBST and incubated with secondary antibody for 1 h at room temperature and signal was detected using iBright FL1000 imaging system (Invitrogen, A32752). The following primary antibodies and concentrations were used: Phospho-p44/42 ERK1/2 (Cell Signaling Technology, 4370, 1:500), p44/42 ERK1/2 (Cell Signaling Technology,4696, 1:1000) and alpha-tubulin (Santa Cruz Biotechnology, sc-32,293, 1:2000). The following secondary antibodies were used: IRDye 800CW goat anti-mouse (LI-COR, 926-32210, 1:1000) and Alexa Fluor 647 AffiniPure goat anti-rabbit (Jackson ImmunoResearch, 111-605-144, 1:1000). ImageJ software (NIH) was used to quantify the blot and calculate the ratio of ppERK1 to ERK1 relative to primary tumor.

### Statistical analysis

Unless otherwise stated, statistical analysis of imaging and wet bench results was performed using Prism 9 (Version 9.5.1 (528); GraphPad Software, LLC; Boston, MA, USA). The number of samples and mice are explicitly stated in methods, text of results, or figure legends with p-values. Data reported with error bars represent mean +/- standard error of the mean (SEM) unless otherwise stated. Fold decrease was calculated by dividing the mean of the control group by the mean of the experimental group. Fold increase was calculated by dividing the mean of the experimental group by the mean of the control group.

## Results

### Niche cell populations and tumor cell phenotypes change during metastatic colonization and disease progression

**Fig. 1:**
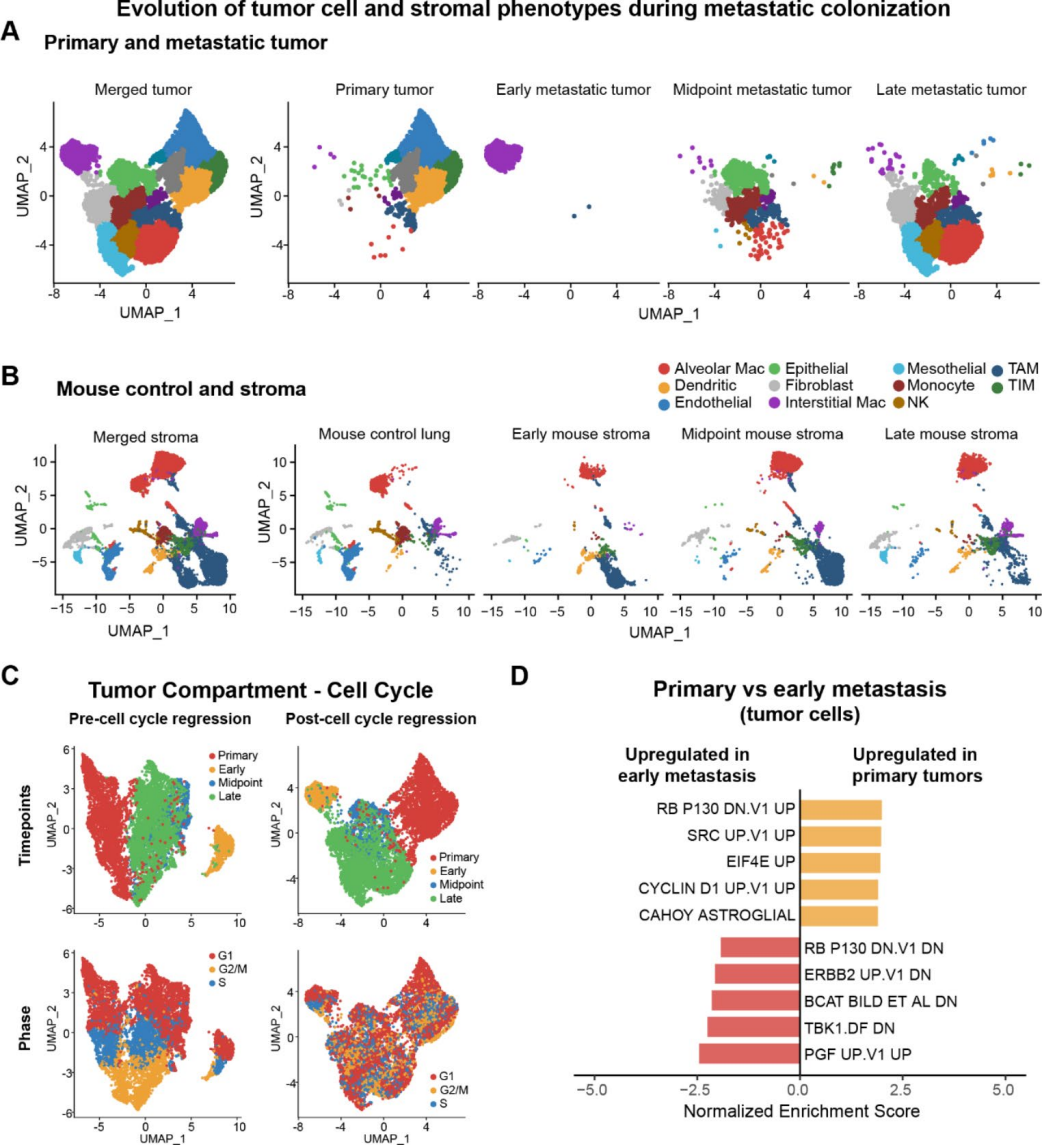
Transition through the lung colonization bottleneck is associated with increased ERK activity. (**A**) and (**B**) UMAP plots showing changes in tumor cell and stromal heterogeneity and phenotypes from those of primary tumors through different stages of metastatic colonization (2, 4, and 7 weeks post-dissemination). Both niche and tumor cell populations evolve substantially as lesions develop. (**C**) UMAP plots the effects of cell cycle on clustering relative to timepoint and the outcome of cell cycle regression (used to generate the plots shown in (**A**)). (**D**) Visualization of results from GSEA comparing transcriptional pathways of tumor cells from a primary tumor and early metastases. Several pathways regulated by MAPK/ERK are increased in early metastases compared to primary tumor

We first sought to determine how osteosarcoma transcriptional profiles changed over the course of disease, as lesions progressed from primary tumor to various stages of metastasis. We reasoned that identification of discrete transcriptional profiles in these contrasting microenvironments could provide insight into mechanisms that facilitate survival, adaptation, and growth of disseminated tumor cells in the lung. To this end, we evaluated scRNAseq data from a primary OS-17 tumor, as well as the slp-mCherry OS-17 tumor cells from early, midpoint, and late time points of metastasis growth (Fig. 1A).

Primary tumors clustered into five groups, suggesting the presence of several distinct tumor cell subpopulations, while early metastatic lesions were less transcriptionally diverse, containing only one cluster when using the same analysis parameters. This early metastasis phenotype was also identified as a rare population within both the primary tumor and the late/established metastatic tumor. In contrast, both the midpoint and late metastases had four clusters of cells, indicating they were comprised of different subpopulations. The same subpopulations were present in both the midpoint and late metastatic timepoints but in different proportions, indicating a relatively high degree of transcriptional similarity between these more established metastases. Three of the four clusters identified in the midpoint and late metastases were also identified in the primary tumor, likewise indicating transcriptional similarity between these timepoints. Cells clustered independently of any cell cycle-related state regardless of timepoint (Fig. 1C). Collectively, these data imply that early metastatic seeding events are associated with a microenvironment-induced adaptative response that begins with a transient, phenotypically homogeneous state, then normalizes to a state of tumor cell heterogeneity more similar to established primary lesions.

To understand what causes disseminated cells to adopt these phenotypes within the lung, we first sought to illuminate how the lung microenvironment evolves during metastatic colonization by isolating tumor-associated stroma using a niche labeling system [53, 55]. Fluorophore secreted from slp-mCherry-labeled OS-17 cells was adsorbed by nearby stroma, allowing identification of niche-associated cells by flow cytometry. We performed scRNAseq on these tumor-associated cells at early (2 weeks after IV inoculation with tumor cells), midpoint (4 weeks), and late (7 weeks) stages of metastatic growth, and on control non-tumor-bearing lungs. We identified stromal cells by clustering the data at high resolution and assigning each cluster to a consensus cell type (Supplemental Fig. 2) These experiments revealed that both the type and number of niche cells varied over time in response to metastasis. Alveolar macrophages showed phenotype shifts in tumor-bearing lung compared to the control lung, but overall, the clusters remained of similar size. As a percentage of total cells, endothelial cells and monocytes were less prevalent in tumor-bearing lung stroma than in control lung. Interstitial macrophages, mesothelial cells, and natural killer (NK) cells formed moderate clusters in the control lung, were nearly absent in the early tumor-associated niche and developed larger clusters in midpoint and late tumor-bearing stroma. Cells identified as tumor-associated macrophages (TAM) were very sparse in the control lung and successively more prevalent in both early and established metastases. Cells identified as tumor-infiltrating macrophages (TIM) were sparse in the control lung and a successively larger number was captured from early to late tumor-bearing stroma. Dendritic cells and fibroblasts remained relatively stable throughout all timepoints, though the phenotype of the fibroblasts evolved over the course of lung colonization (Fig. 1B).

### MAPK-driven signatures are overrepresented in early metastases compared to primary and advanced metastatic lesions

To identify pathways activated in cells that survive dissemination, we identified genes differentially expressed in early metastatic tumors relative to primary tumor. We performed gene set enrichment analysis (GSEA) of these genes against the transcriptional outputs of validated oncogenic pathways (C6 signatures of MSigDB) (Fig. 1D) [75, 76]. We found that pathways involving JNK, JAK2, PDGF, VEGFA, and ERBB2 activity were all comparatively upregulated in early metastatic lesions; all of these proteins mediate or are mediated by the MAPK/ERK pathway [74]. These findings are consistent with previously reports suggesting that MAPK-driven programs might function as important regulators of osteosarcoma metastatic competence [12].

**Fig. 2:**
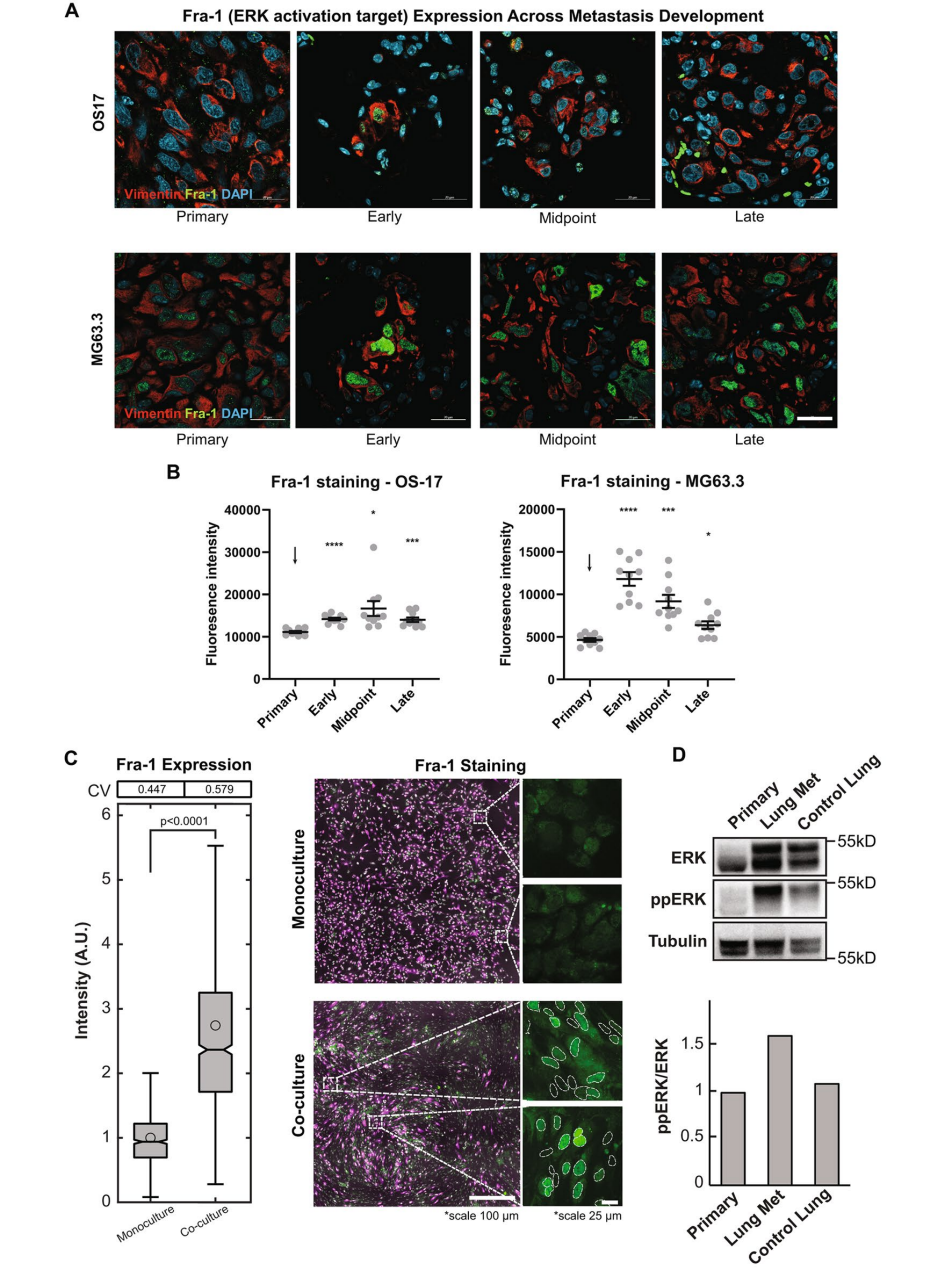
ERK phosphorylation and Fra-1 expression are altered by the microenvironment. (**A**) Representative IHC-F images of expression of Fra-1 (green) in human cell line tumors (vimentin, red) at various time points. Counterstained with DAPI (blue). In both cell lines, metastases express more Fra-1 compared to primary tumors. This is consistent with the OS-17 scRNAseq data. Scale bar, 20 μm. Ten images per time point were captured. (**B**) Quantification of fluorescence intensity across samples stained for Fra-1; ten images per time point were captured and analyzed. When conditions are compared using one-way ANOVA with Dunnet’s T3 multiple comparisons, p values are shown above the comparison conditions (* p < 0.05, ** p < 0.01, *** p < 0.001, and **** p < 0.0001 relative to the control condition, denoted by the arrow). (**C**) Box plot (left) showing Fra-1 expression in OS-17 reporter cells in monoculture, and when co-cultured with monocytes and Normal Human Lung Fibroblasts. Coefficient of variation (CV) is listed. Microscopy images (right) show OS-17 reporter cells (magenta) with Hoechst stain (white) and Fra-1 (green) in monoculture and in co-culture with monocytes and Normal Human Lung Fibroblasts. Insets show only Fra-1 staining. Dashed circles outline OS-17 nuclei in co-culture conditions. Tumor cells express more Fra-1 when monocytes and fibroblasts are present. Monoculture and co-culture were performed in triplicate for each experiment, and two independent experiments were performed. (**D**) Total and phosphorylated ERK1/2 were measured via Western blot in tissue from OS-17 primary tumor, midpoint metastasis, and control lungs. Phosphorylated ERK is increased in metastatic lesions compared to the primary tumor and control lungs

To confirm that MAPK/ERK pathways are activated at a protein level, we evaluated primary tumors and metastatic lesions from early, mid, and late time points of disease in both OS-17 and MG63.3 cell lines by immunofluorescence staining of Fra-1 (the protein product of the gene *FOSL1*) (Fig. 2A, B, Supplemental Figs. 3–4). Fra-1 is an AP1 transcription factor and a well-established downstream target of MAPK/ERK that functions as a linear integrator of ERK signaling activity over time [77]. Thus, total Fra-1 expression provides an accurate readout of both the magnitude and duration of ERK signaling of single cells within their local microenvironment. Consistent with the scRNAseq results, we found that Fra-1 was expressed at uniformly low levels in primary tumors of both OS-17 and MG63.3 human cell lines, whereas in metastases Fra-1 was strongly but heterogeneously expressed (Fig. 2A, B). Western blotting of primary tumor, control lungs, and midpoint metastasis-bearing lungs showed that ERK1/2 phosphorylation was greatest in metastasis-bearing lungs (Fig. 2D).

To gain insight into this process, we co-cultured primary human monocytes and lung fibroblasts with OS-17 cells and stained for Fra-1. Consistent with staining of early metastasis bearing mice, co-cultured OS-17 cells exhibited a significant (p < 0.0001), 2.7-fold increase in Fra-1 expression, with a high level of expression variability regionally from cell-to-cell, relative to monocultures (Fig. 2C). These results suggest that Fra-1 expression, and cell-to-cell expression heterogeneity, is driven by ERK-activating signals in OS-17. These signals likely emanate from tumor-associated stromal cells. One can infer that heterogenous ERK pathway activation in early metastatic lesions could arise through cell-autonomous mechanisms, spatial variation in signals available to tumors cells within the metastatic microenvironment, or both [78]. In established metastases (midpoint and late), a smaller proportion of tumor cells expressed high levels of Fra-1, consistent with the scRNAseq data and suggesting a return to primary tumor-like transcriptional state over time (Figs. 1A and 2A). Together, these data indicate a distinct biological and temporal association between activation of MAPK/ERK signaling networks in the establishment of successful metastatic lesions in the lung.

### Niche cell-derived growth factors drive ERK activity in early metastatic cells

**Fig. 3:**
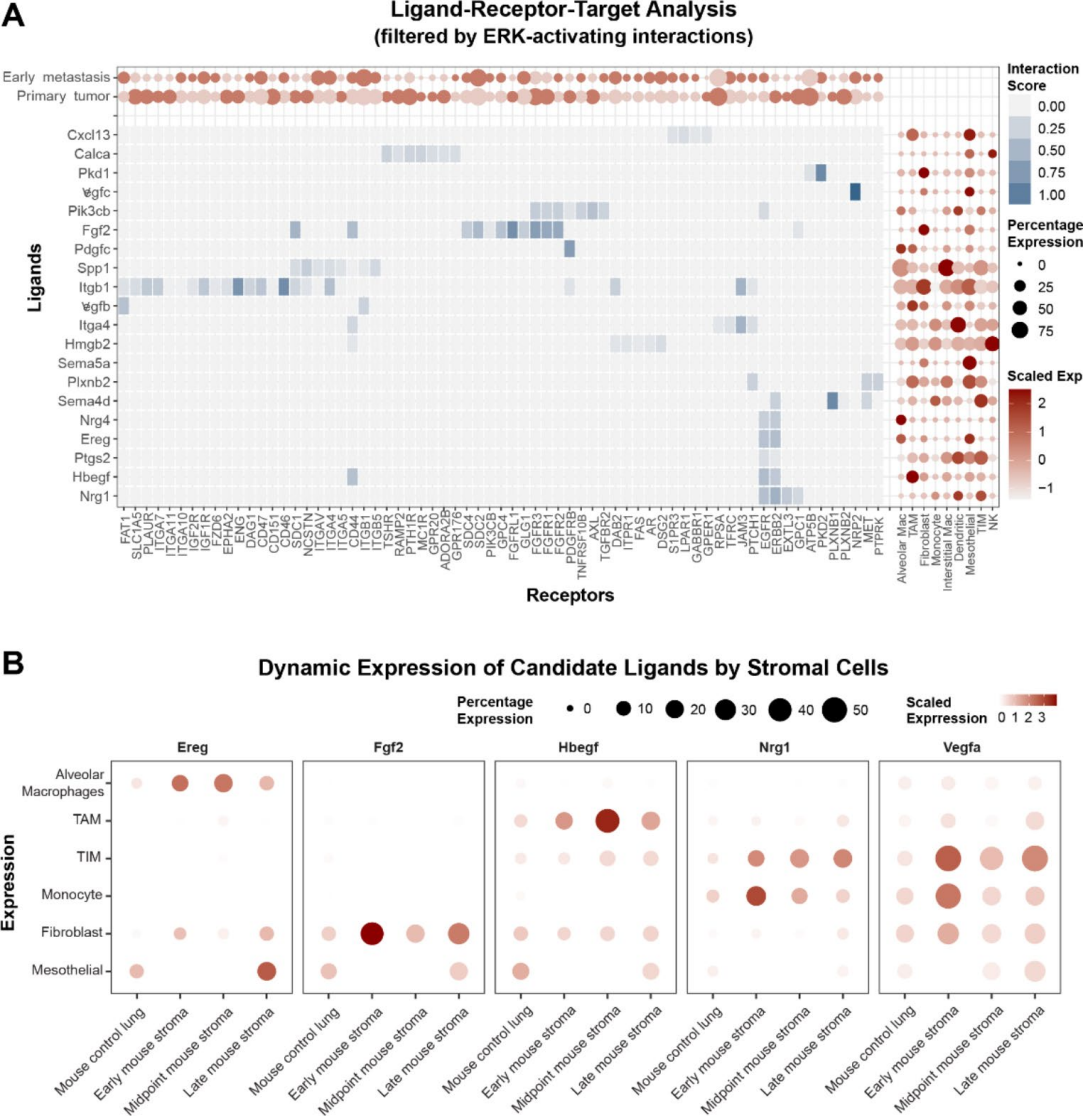
Host cells produce specific growth factors predicted to drive ERK activity in osteosarcoma cells. (**A**) NicheNet analysis showing ligand/receptor pairs predicted to regulate the elevated MAPK state seen in early metastases compared to primary tumor. (**B**) Dot plot showing transcription of growth factors in control lungs and metastasis-bearing lungs separated by growth point. A variety of cell types transcribed growth factors. Most growth factors were transcribed more highly, and by more cells, in lungs bearing metastases

We next wanted to understand what specific ligands from niche cells may drive the comparatively increased ERK activity in early metastatic lesions. To this end we utilized NicheNet, a computational algorithm designed to use scRNAseq data and existing literature to predict ligand-receptor interactions that drive specific gene expression changes within complex biological systems [72]. For our analysis, we identified the target gene set as those genes differentially expressed in early metastatic lesions relative to primary tumors (via GSEA, above) and used NicheNet to predict what stromal ligand/tumor receptor pairs may drive these changes (Fig. 3A). This analysis identified several ligand-receptor pairs that canonically signal through activation of MAPK/ERK: VEGFC, FGF2, PDGFC, EREG, HBEGF, and NRG1. These growth factors’ corresponding receptors exhibited significant shifts in expression within primary and metastatic tumor cells, possibly suggesting a role in adaptation. NRP2, FGFR2, GLG1, GPC4, PIK3CB, CDC2, GPR176, CD44, and EGFR were more highly transcribed by early metastatic cells, while the transcription of PDGFRB, GPC1, FGFR1, FGFR3, FGFRL1, SDC1, and ERBB2 was higher in primary tumor cells.

Respecting that the NicheNet algorithm does not incorporate magnitude of ligand transcription when predicting ligand-pair interactions, and that the positive “hits” in this assay could have arisen from multiple potential ligands that activate these pathways, we further evaluated niche-specific ligand expression using a focused list of growth factors known to regulate ERK activity (Supplemental Fig. 5) [74, 79, 80]. We used this list in concert with the scRNAseq data from niche cells in the control and metastasis-bearing lungs to determine what growth factors were more highly transcribed when metastases were present compared to control lungs, and what niche cell populations transcribed these growth factors (Fig. 3B). We found that the majority of growth factors were more highly transcribed in metastasis-bearing lungs compared to control lungs (Supplemental Fig. 5). We found that factors such as TGFβ1 and VEGFA were highly transcribed throughout the metastatic process by numerous stromal cell types. In contrast, other growth factors (such as FGF2 and FGF10) were predominantly produced by fibroblasts (Fig. 3B, Supplemental Fig. 5B). We also found that the percentage of cells transcribing growth factors was increased in metastasis-bearing lungs compared to control lungs. This indicates that changes in growth factor expression were not due solely to changes in cell numbers, but cell transcriptional behavior as well (Supplemental Fig. 6).

### Identified niche growth factors heterogeneously induce osteosarcoma ERK signaling and downstream target expression

**Fig. 4:**
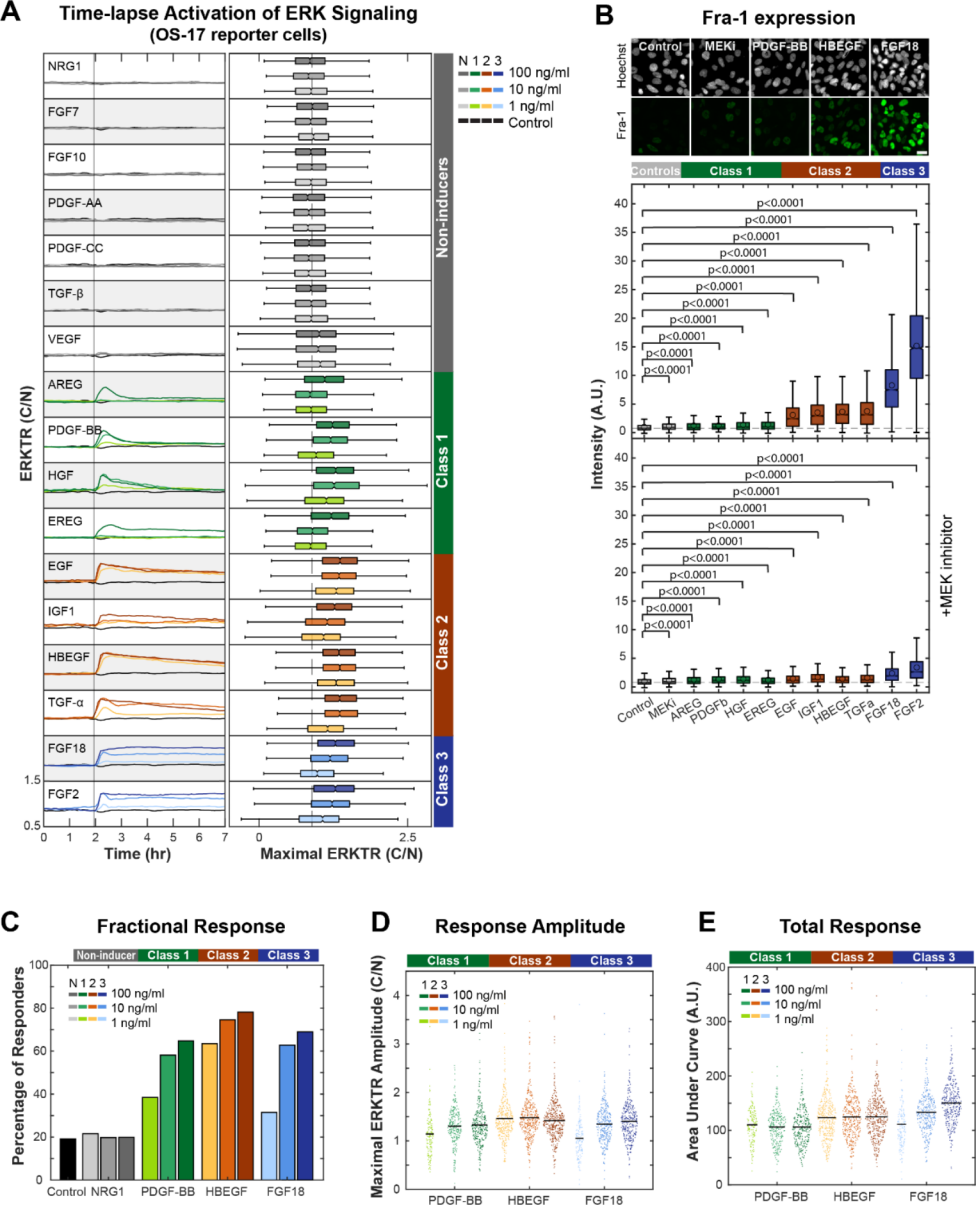
Osteosarcoma cells respond to growth factors with increased ERK phosphorylation and activity. (**A**) Mean traces (left panel) showing ERK activity of OS-17 reporter cells in response to growth factor addition at different concentrations. Each trace shows mean ERK activity over time obtained through measurement of nuclear to cytoplasmic fluorescence ratio of > 5000 cells. Box plots (right panel) depicting maximum ERK activity per cell following growth factor addition at different concentrations. Growth factors are grouped according to their proposed response groups. Dashed line indicates median of control treated cells. Three independent replicate experiments were performed, with a minimum of 500 cells per condition in each experiment. (**B**) Box plot showing fluorescence intensity of Fra-1 staining in OS-17 cells exposed to various growth factors. Of the growth factors that noticeably increased ERK phosphorylation, EGF, FGF2, FGF18, HBEGF, IGF1, and TGFα noticeably increased Fra-1. Co-treatment with a MEK inhibitor decreased Fra-1 expression to near baseline except when cells were treated with FGF2 and FGF18. Dashed line indicates median of control treated cells; circles indicate mean intensity. Three independent replicate experiments were performed, with a minimum of 1,000 cells per condition in each experiment. (**C**) Bar graph showing the percentage of cells that responded with increased ERK phosphorylation when treated with the listed growth factors. Response to NRG1, classified as a non-inducer, was equivalent to the control at all doses. The other growth factors responded in a dose-dependent manner, with HBEGF (Class 2) showing the greatest percentage of responding cells. A minimum of 400 total cells per conditions were used for the analysis from three independent replicate experiments. (**D**) Violin plot showing the maximal ERK amplitude in response to listed growth factors. The maximal amplitude is dose-dependent in PDGF-BB and FGF18. A minimum of 400 total cells per condition were used for the analysis from three independent replicate experiments. (**E**) Violin plot showing the area under the curve of each cell as a proxy for duration of ERK phosphorylation in response to listed growth factors. The duration of response is dose-dependent only in cells treated with FGF18. A minimum of 400 total cells per condition were used for the analysis from three independent replicate experiments

To quantitatively evaluate the effects of niche growth factor expression on osteosarcoma cell signaling, we generated OS-17 cells stably expressing the fluorescent ERK translocation reporter (ERKTR), hereafter referred to as “reporter cells” [81]. The ERKTR reporter translocates between the cytoplasm and nucleus based on instantaneous changes in ERK signaling state, which allows for quantitative measurement of single cell ERK response to varying concentrations and compositions of microenvironmental growth factors using live-cell imaging (Supplemental Fig. 7A). Based on the NicheNet data and transcriptional data shown in the dot plot (Fig. 3), we chose to further investigate the effects of AREG, EGF, EREG, HBEGF, NRG1, TGFα, FGF2, FGF7, FGF10, FGF18, HGF, IGF-1, PDGF-AA, PDGF-BB, PDGF-CC, TGFβ1, and VEGFα on osteosarcoma cells in vitro alone or in combination with the highly selective MEK inhibitor (MEKi) PD0325901. Using the reporter cells, we found that exposure to HBEGF, FGF2, FGF18, PDGF-BB, HGF, IGF-1, EREG, AREG and TGFα, which were overexpressed in the metastatic lung microenvironment, produced a dose- and growth factor-specific increase in ERK activity (Fig. 4A). This was reflected in significant (p < 0.0001) changes in Fra-1 staining (up to 15-fold increase) and was abrogated by co-treatment with MEKi (Fig. 4B). The growth factors could be grouped into three distinct “Classes” based on response duration: Class 1, which elicited transient responses (HGF, PDGF-BB, AREG and EREG); Class 2, which elicited prolonged but gradually waning responses (EGF, HBEGF, TGFα, and IGF1); and Class 3, which elicited sustained responses (FGF2 and FGF18).

Within each growth factor Class we observed variable potency (Fig. 4A). Some growth factors, such as HBEGF, produced high amplitude responses at low concentrations (1 ng/ml), while others such as EREG, only elicited responses at higher, likely supra-physiological, concentrations. Somewhat surprisingly, OS-17 cells were insensitive to the growth factors PDGF-AA, PDGF-CC, FGF10, and NRG1, even at supraphysiological concentrations (100 ng/mL), which have been reported as having biological importance in osteosarcoma [82–86]. This could represent model-specific or cell culture-related effects.

We next chose to explore how these growth factors might induce heterogeneous ERK and target gene responses [78, 87]. To this end, we measured the fractional response i.e., the number of cells in a population that respond to a uniform concentration of growth factor at any given time (Fig. 4C). We found that the Class 2 ligand, HBEGF, produced a substantial fraction of responding cells even at the lowest concentration of growth factor tested (> 60% responders, 1 ng/mL), indicating a high probability that an individual tumor cell will respond if exposed to this ligand even at relatively low concentrations. Class 1 and 3 growth factors PDGF-BB and FGF18 had comparatively lower, dose-dependent fractional responses, suggesting that the probability of an ERK response in any individual cell was more closely associated with ligand abundance compared to Class 2 ligand HBEGF (Fig. 4C). We also found that the intensity of response was dose-dependent in PDGF-BB and FGF18, but not HBEGF, which was determined by evaluating the response amplitude and the area under the curve i.e. average ERK activity over time (Fig. 4D, E). These results suggested that exposure to ligands that induce these different classes of ERK-activating responses should induce different patterns of target gene responses. For instance, the probability of an FGF18-induced single cell ERK response, extrapolated from percent responder analyses, was dose dependent (Fig. 4C) and of high intensity and long duration when it occurred (Fig. 4D, E). In the context of Fra-1 as a linear integrator of ERK activity, these FGF18 characteristics predict both a high level of cell-to-cell Fra-1 heterogeneity and a high relative level of expression in responding cells, which was observed in our experimental data (Fig. 4B, Supplemental Fig. 8A). In contrast, Class 1 (e.g. PDGF-BB) and Class 2 (e.g. HBEGF) would be predicted to express Fra-1 less intensely (up to 1.4-fold increase for Class 1, 3.7-fold increase for Class 2 and 15-fold increase for Class 3) and less heterogeneously based on ligand-induced ERK response fraction, and response magnitude/duration characteristics as was observed in our data (Fig. 4, Supplemental Fig. 8A, B).

We reasoned that if the mechanisms of cell-to-cell ERK target gene heterogeneity are rooted in ligand-response properties, then constitutive ERK activation should abolish such variation. Indeed, staining for Fra-1 in RAS-mutant 143B osteosarcoma cells showed near uniform levels of Fra-1 expression in single cells, and no evidence of heterogeneity or enhanced expression in the presence of exogenous FGF18 (Supplemental Fig. 9B).

Taken collectively, these data imply that ligands within the microenvironment—in particular FGFR ligands—play an important role in osteosarcoma ERK activation. These data also suggest a potential mechanism for the observed Fra-1 heterogeneity observed with in vivo murine tumors (Fig. 2A).

### Metastases show ERK-promoted MCL1 signaling

**Fig. 5:**
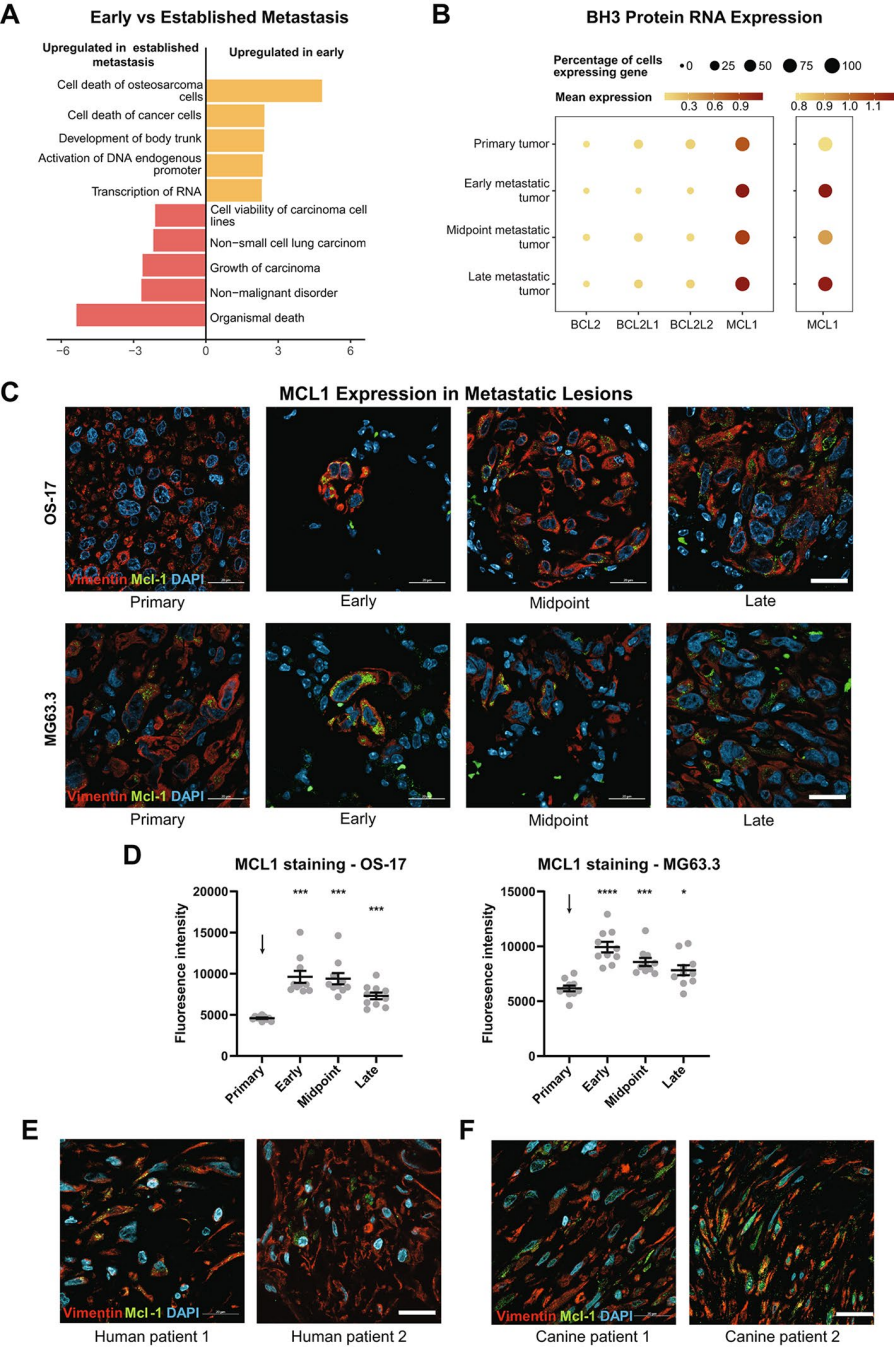
Early experimental metastases display ERK-promoted MCL1 signaling. (**A**) Visualization of IPA results examining transcriptional pathways upregulated in tumor cells from early metastases compared to established (midpoint and late) metastases. Early metastases have upregulation of cell death pathways, consistent with the hostility of the early metastatic environment. (**B**) Dot plot of scRNAseq data from OS-17 tumors at progressive disease timepoints evaluating transcription of anti-apoptotic proteins. MCL1 is the predominant anti-apoptotic protein at all timepoints (left). When scaled to better differentiate transcription levels (right), MCL1 is shown to be highly transcribed by early metastases, and all metastatic tumor timepoints transcribe MCL1 more than primary tumor.​ BCL2L1 is also known as BCLXL and BCL2L2 is also known as BCLW. (**C**) Representative IHC-F images of expression of MCL1 (green) in human cell line tumors (vimentin, red) at various time points. Counterstained with DAPI (blue). In OS-17, early metastases express the most MCL1 compared to primary tumors and later metastases, consistent with scRNAseq data. Tumors grown from MG63.3 cells show a similar pattern. Ten images per time point were captured. (**D**) Quantitative analysis of MCL1 expression from immunofluorescent stains of multiple biological replicates as stained above. When conditions are compared using one-way ANOVA with Dunnet’s T3 multiple comparisons, p values are shown above the comparison conditions (* p < 0.05, ** p < 0.01, *** p < 0.001, and **** p < 0.0001 relative to the control condition, denoted by the arrow). (**E**, **F**) Osteosarcoma metastases (vimentin, red) from two different human (E) and two different canine (F) patients express MCL1 (green). Images counterstained with DAPI (blue). One image per patient sample was captured

To determine which cell processes and behaviors might be changed by ERK activity during metastatic colonization, we utilized IPA to identify gene expression programs that are activated in early vs. established metastases (combination of midpoint and late timepoints) (Fig. 5A) [74]. We found that early metastases had a noticeable increase in pathways that regulate cell death vs. survival. This led us to ask whether ERK signaling in early metastatic lesions is associated with a corresponding upregulation of anti-apoptotic proteins that may favor tumor persistence and growth in the hostile lung environment [32, 88–101]. Consistent with this hypothesis, we found that multiple BCL2 proteins are transcribed in the primary tumor and early, midpoint, and late metastases, with MCL1 being significantly more abundant compared to other anti-apoptotic factors regardless of the time point (Fig. 5B). We also found that MCL1 was transcribed more in metastatic tumors than the primary tumor.

To confirm that MCL1 expression in vivo was consistent with the scRNAseq data, we performed IHC-F on primary tumors and metastases from early, midpoint, and late metastases (Fig. 5C, Supplemental Figs. 10–11). We evaluated lesions in both OS-17 and MG63.3 cell lines. Consistent with the scRNAseq data, metastases in both cell lines had higher MCL1 expression than the primary tumor regardless of the metastatic time point. Interestingly, early metastases showed the highest amount of MCL1 expression in both cell lines, with later metastases showing comparatively decreased MCL1 staining (Fig. 5D). The intensity of expression of MCL1 in both cell lines was overall congruous with Fra-1 expression—that is, low in the primary tumor, with elevated, heterogeneous expression in metastases (Figs. 2A-B and 5C-D).

With an eye toward translation, we wanted to validate that pronounced MCL1 expression is seen in spontaneous, established metastases. To accomplish this, we performed immunofluorescent staining on sections of formalin fixed, paraffin embedded lung metastases from human osteosarcoma patients. Both specimens showed unequivocal expression of MCL1 within metastatic tumor cells (Fig. 5E). As pet dogs often develop highly metastatic osteosarcoma, and as integrative trials present opportunities to accelerate translation, we also wanted to confirm that metastatic canine osteosarcomas also show prominent MCL1 expression [42–44, 47–49, 102–109]. Slides from formalin fixed, paraffin embedded canine metastases were similarly stained. Of the two patients evaluated, all showed clear expression of MCL1 in metastases (Fig. 5F).

**Fig. 6:**
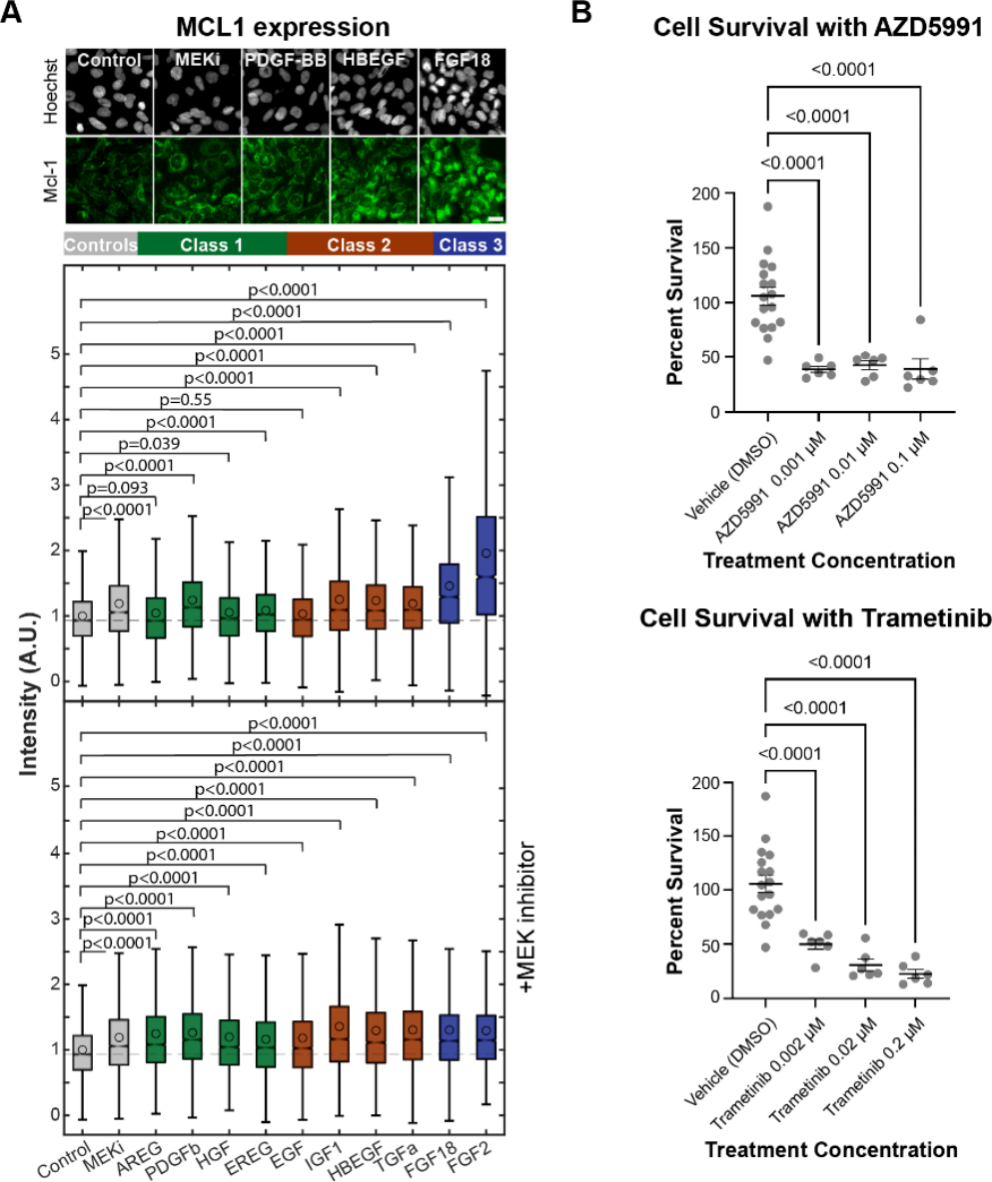
Osteosarcoma cells are vulnerable to MAPK and MCL1 inhibition in vitro. (**A**) Fluorescence intensity of MCL1 staining in OS-17 cells exposed to various growth factors. Of the growth factors that increase ERK phosphorylation, only FGF2 and FGF18 noticeably increase MCL1 expression. Co-treatment with a MEK inhibitor abrogated or reduced increases in MCL1 expression in most growth factors.​ Dashed line indicates median of control treated cells; circles indicate mean intensity. (**B**) Dot plot showing the “percent survival” of spheroids after each of various treatment conditions. The number of metastatic organoids after 72 h of treatment was divided by the pre-treatment amount to give percent survival, then compared via ANOVA; multiple comparisons were corrected for by controlling the False Discovery Rate using the original method of Benjamini and Hochberg. Treatment with trametinib or AZD5991 greatly reduced the survival of metastatic organoids, even at low concentrations. Two independent experiments were performed, and in each experiment vehicle control was performed in sextuplicate, and each treatment condition was performed in triplicate

Having established that MCL1 is expressed in patient metastases and at variable levels throughout an in vivo murine model of disease (where its expression generally correlated with ERK activity), we next sought to determine if identified growth factors were sufficient for increased MCL1 protein expression and if MEKi abrogated this response. We found that exposure to FGF2, FGF18, HBEGF, IGF1, PDGF-BB, and TGFα significantly (p < 0.0001) increased MCL1 expression in tumor cells (by at least 1.2-fold and up to 2-fold) and treatment with the MEKi reduced this response (Fig. 6A). It is worth noting that all cells treated with MEKi (alone or in combination with growth factors) showed a modest (average increase of 1.3-fold, up to 1.4-fold) but statistically significant (p < 0.0001) increase in MCL1 staining compared to control, likely due to the mildly cytotoxic effects of MEKi and/or activation of parallel regulatory pathways capable of influencing MCL1 expression [33, 110–114]. Nonetheless, the magnitude of increase in MCL1 expression directly correlated with the ERK response characteristics, with Class 3 growth factors producing the greatest intensity of staining for MCL1 (average of 1.7-fold increase), and Class 2 and Class 1 factors producing less intense MCL1 staining (average increase of 1.2-fold and 1.1-fold respectively). These results were similar to the results obtained for Fra-1 (Figs. 4A and B and 6A). These results confirmed that many—but not all—identified growth factors in the metastatic niche increase ERK activity and are largely sufficient to increase MCL1 expression in osteosarcoma.

To further confirm the connection between ERK activity and MCL1 expression in the metastatic lung microenvironment we utilized 143B cells, which harbor a *KRAS* mutation that confers persistent ERK pathway activation (Supplemental Fig. 8B) [115, 116]. We found that 143B cells treated with FGF18 alone had unchanged Fra-1 expression and marginally decreased MCL1 expression (likely due to activation of negative feedback loops). Co-treatment with MEKi resulted in nearly complete elimination of Fra-1 expression and significant reduction in MCL1 expression, further solidifying ERK’s role in MCL1 expression.

We next wanted to investigate if the MAPK pathway and/or MCL1 were therapeutically targetable dependencies. To do this, we first utilized a metastatic organoid model in vitro (Supplemental Fig. 12). Metastatic organoids significantly (p < 0.0001) reduced in number compared to control when treated with the MEK inhibitor trametinib (up to 4.6-fold decrease), or the MCL1-specific BH3 mimetic AZD5991 (up to 2.7-fold decrease), even low concentrations (Fig. 6B).

### Metastases are vulnerable to MCL1 inhibition in vivo and exhibit synergy in combination with chemotherapy

We next wanted to determine if early osteosarcoma metastases are dependent on MAPK signaling and/or MCL1 in vivo. To this end, we first performed a small pilot study to evaluate tolerability and optimize dosing of AZD5991 and trametinib. Administering AZD5991 or its vehicle IV resulted in tail vein necrosis due to the high viscosity of the solvents; all subsequent doses were given IP. H&E sections of lungs showed that treatment with AZD5991, but not trametinib, reduced the number of metastases and metastatic burden (percentage of lung area colonized by tumor) in a dose-dependent manner (Supplemental Fig. 1).

Knowing that MCL1 increases a cell’s resistance to apoptosis and that, clinically, metastatic lesions often demonstrate resistance to conventional chemotherapies, we wanted to determine whether inhibiting MCL1 might restore chemo-sensitivity [117, 118]. Recognizing that many patients with non-resectable metastatic osteosarcoma are treated with ifosfamide, we chose to use cyclophosphamide as our chemotherapeutic agent. Cyclophosphamide is an in-class relative to ifosfamide, has shown activity in osteosarcoma lung metastases in patients, and is better tolerated in mice than ifosfamide [119–126]. Due to the results of the pilot study (Supplemental Fig. 1), we chose to exclude trametinib from this experiment.

We hypothesized that co-treatment with AZD5991 and cyclophosphamide would result in significant reduction in the number and size of metastases while treatment with AZD5991 alone would result in a modest decrease. To test this, we tail vein injected mice with luciferase expressing OS-17 cells (OS-17-FLUC). Mice were randomized by luciferase luminescence one week prior to start of treatment to account for differences in tumor uptake. Mice were also imaged weekly throughout treatment until endpoint. Treatments began four weeks after injection—a timepoint that corresponds to “midpoint” established metastases in OS-17 cells. This time point was chosen due to the sustained transcription of MCL1 in metastases of all timepoints (Fig. 5B), presence of MCL1 in established patient tumors (Fig. 5E, F), and to model the fact that salvage therapy is usually initiated in patients after metastases are detectable. Endpoint was established once the first mouse in any group met established humane endpoint criteria (clinical symptoms) and had metastatic lesions confirmed at necropsy; this occurred 46 days after tail vein injection of tumor cells. At this point, mice in all treatment groups were humanely euthanized and lungs were collected for staining and evaluation by IHC-F.

**Fig. 7:**
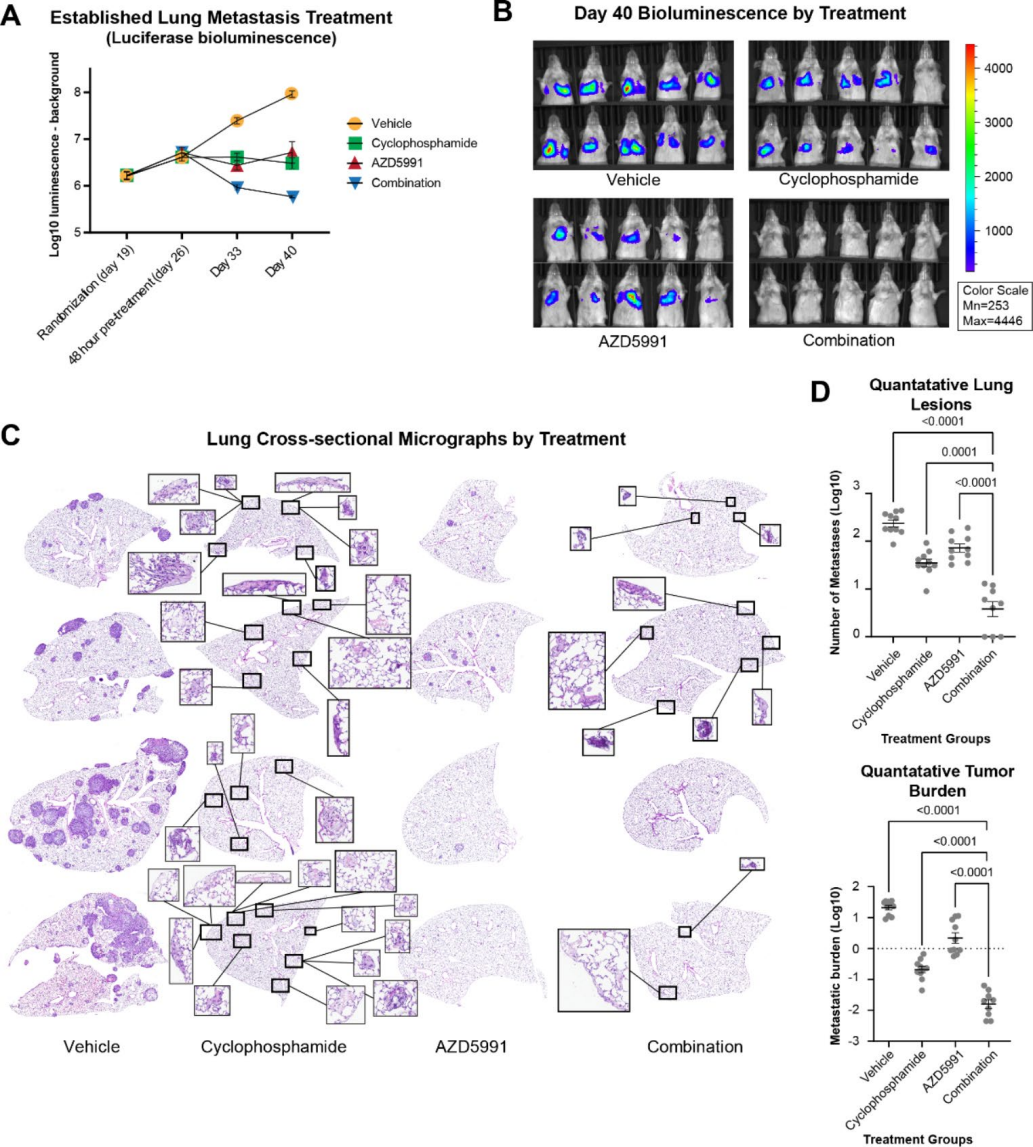
Osteosarcoma metastases are vulnerable to MCL1 inhibition in vivo. (**A**) Dot plot showing mean + SEM of Log10 luciferase luminescence minus background of each study group at timepoints throughout the study; all forty mice were imaged once on the listed days. Cyclophosphamide and AZD5991 groups had similar reductions in luminescence compared to the vehicle group. The combination treatment group had marked reduction in fluorescence. (**B**) Images of treatment groups taken six days before endpoint criteria were met showing luminescence of tumor cells after IP injection of luciferin. All images are scaled to the same minimum and maximum (right). All mice in the dual treatment had undetectable metastases. (**C**) Representative H&E sections showing lung sections from each study group. Metastases in the combination treatment group were drastically reduced, with two of the shown lung sections containing no metastases.​ Inserts showing an additional 10x magnification are included in the cyclophosphamide and combination micrographs to show identified lesions, as these are not easily seen at lower magnification. (**D**) Dot plot showing the number of metastases, and metastatic burden, of mouse lungs from each treatment group. These values were quantified in three lung sections per mouse, from all 10 mice in each treatment group, and compared via one-way ANOVA with post-hoc multiple comparisons relative to the combination treatment group. The metastatic burden was calculated dividing the area of all tumors by the area of the lung lobe and multiplying by 100. Treatment with AZD5991 resulted in noticeable reduction of number of metastases and metastatic burden. Combining AZD5991 and cyclophosphamide resulted in drastic reduction in the number of metastases and metastatic burden; one mouse in this group had no detectable metastases

Throughout treatment, luciferase luminescence in the combined treatment group decreased the most drastically, while single-agent groups maintained a luminescence similar to pre-treatment levels (Fig. 7A). The final luciferase images, scaled to the vehicle control group, showed no detectable luminescence within the combination treatment group (Fig. 7B). Further evaluation of lung sections collected from each mouse showed that treatment with AZD5991 alone noticeably, significantly (p = 0.0001) reduced both the number of metastatic lesions per Sect. (3.1-fold decrease) and the overall metastatic burden, defined as the area percentage of each lung section affected by tumor, (5.7-fold decrease). In comparison, cyclophosphamide significantly (p < 0.0001) reduced number of metastases (6.5-fold decrease) and metastatic burden (89.4-fold decrease). Treatment with both AZD5991 and cyclophosphamide resulted in drastic, significant (p < 0.0001) reductions in metastasis number (50.9-fold decrease) and volume (1,125-fold decrease) compared to vehicle and single drug treatment groups (Fig. 7C, D). To evaluate for an additive vs. synergistic effect of cyclophosphamide and AZD5991, the log-transformed data (to preserve normality) were also analyzed using an ordinary two-way ANOVA, which revealed an interaction for both lesion number (p = 0.03) and tumor burden (p = 0.05), suggesting a more-than-additive effect of the combination treatment.

## Discussion

Herein, we used scRNAseq, immunofluorescence microscopy, live cell imaging of tumor cells engineered for real-time reporting of signaling activity, and murine models of metastasis to elucidate microenvironmental cues that promote osteosarcoma cell survival during lung colonization. Our data suggest that several growth factors produced by tumor-associated stromal cells within the developing metastatic niche drive ERK activity and increased MCL1 expression in subsets of osteosarcoma cells, and that both ERK activity and MCL1 expression are particularly elevated in early metastases. We showed that MCL1 is the dominant anti-apoptotic factor facilitating survival within the early metastatic niche, that expression of MCL1 persists in established metastases, and that established metastases are vulnerable to pharmacological disruption of MCL1 activity.

Of particular interest, an MCL1-specific inhibitor, AZD5991, markedly reduced metastatic colonization of osteosarcoma cells. When combined with conventional chemotherapy agents, this MCL1 inhibitor showed strong anti-tumor activity in mice with established metastatic disease, even eliminating detectable disease in some mice. In addition, we showed that osteosarcoma cells evolve transcriptionally as disease progresses from primary tumor to disseminated tumor cells to established metastases, and that lung colonization appears to involve stepwise, progressive changes in both tumor and stromal cell behavior. Intriguingly, we found that early metastases were transcriptionally homogenous and distinct from primary tumor and established metastatic lesions, and that populations similar to those present in the primary tumor re-emerged as metastases became more established.

As metastatic lesions develop, both the transcriptional phenotypes and the composition of tumor-associated stromal cells evolve. Marked changes in fibroblasts, mesothelial cells, NK cells, and macrophage populations alter the signaling environment in ways that are likely to affect tumor cells far beyond the signaling pathways that became the focus of this manuscript. These overall changes in the stromal microenvironment and the re-emergence of primary tumor cell populations in established metastases suggest that tumors remodel the surrounding environment to one that, in some ways, resembles the primary tumor.

When evaluated by IHC-F, MCL1 expression appears to peak in disseminated cells, subsequently becoming somewhat attenuated with disease progression. Indeed, established metastases may require less MCL1 to survive, as the stresses of the established niche are likely far less than those experienced in the early lung. Perhaps by returning the microenvironment to a more hostile, “pre-remodeling” state, established metastases could be made even more susceptible to MCL1 inhibitors and conventional chemotherapeutics. Alternatively, the early metastatic niche may select for tumor cell subpopulations that respond most strongly to ERK-activating signals present within that environment. Indeed, our experiments show a significant degree of heterogeneity in the ERK, ERK target gene, and MCL1 responses within tumor cell populations, both in vitro and in vivo. The precise mechanisms and selective pressures behind osteosarcoma’s dependence on MCL1 are an exciting avenue of future research.

We showed that numerous different populations of host cells produced a wide variety of growth factors that drove ERK activity and MCL1 expression. Many of these cell populations, such as mesothelial cells, are not typically thought to produce growth factors in the context of tumor growth. Unfortunately, attempts to target growth factors and their downstream pathways by inhibiting receptor tyrosine kinases have been only modestly successful in osteosarcoma [127]. A significant reason for this is the lack of specificity of growth factors and their receptors. In osteosarcoma clinical trials, the most successful drugs have been those that target multiple receptor tyrosine kinases, however, these often result in dose-limiting, systemic toxicities [3, 117, 127–129]. Additionally, patient-to-patient and even cell-to-cell heterogeneity exists in receptor tyrosine kinase expression, potentially limiting their application even further [13, 21–27, 130, 131]. Our data showed that osteosarcoma likely displays spatial and temporal variability in ERK activity on a cell-to-cell level. Indeed, the environmental variations in growth factor source, composition and transcription levels that we showed were present at various time points in metastases could have profound effects on ERK signaling magnitude and duration. This may be why MEK inhibition was effective at killing metastatic organoids, which exist in a relatively static environment in vitro. In contrast, the variations in growth factors and ERK activity present in in vivo metastases may have limited the efficacy of MAPK inhibition. Interestingly, we found that AZD5991 showed maximal effect even at the lowest dose.

Our data imply that while MAPK/ERK promote MCL1 expression, it is not the sole promoter. This is consistent with existing literature showing that MCL1 may be upregulated by IL6, VEGF, the Notch-1 signaling pathway, the Akt and STAT-3 pathways, and others [32]. Indeed, the P13K/Akt pathway (downstream of EGFR and others) was shown to likely be altered in early metastases via GSEA-KEGG. Activation of the Akt pathway and subsequent MCL1 upregulation could help explain the lack of trametinib efficacy in vivo. The MAPK pathway is also known to exert a moderate level of negative feedback on the Akt pathway, which could explain the slight increase in MCL1 expression in cells treated with a MEKi in vitro: Akt activity (and MCL1 expression) would presumably increase upon release of negative feedback from MAPK [132, 133]. Further illumination of this and other upstream regulators of MCL1 and its potential additional roles in osteosarcoma beyond promoting survival in the metastatic niche may be a rich vein of future research [32–35].

The use of an immunocompromised mouse strain bearing human tumors must be considered as a limitation of our study. While using a syngeneic model would have allowed for fuller evaluation of tumor-host interactions, these tumors are still fundamentally of murine origin and therefore meaningfully different from human disease [134]. An additional limitation of our study was the use of an experimental metastasis model that does not account for the prior steps of metastasis–only the final ones of lung colonization. Although our model does not account for these processes preceding colonization (detachment from primary tumor, etc.), studies have shown that spontaneously occurring tumors release millions of tumor cells into the blood stream or lymphatics, indicating that our tail vein model of metastasis is reasonably representative of the colonization process [135, 136]. Indeed, we chose this model because colonization was precisely what we wished to evaluate. Further studies including primary tumors and spontaneous metastasis to more fully evaluate the role of MCL1 in other aspects of the metastatic process may be pursued in the future.

Confirming expression of MCL1 in human osteosarcoma metastases and spontaneous canine osteosarcoma metastases argues for further testing of MCL1 inhibitors in canine cell lines and eventually canine clinical trials as a vetting process prior to human clinical trials. Such trials need not be limited to AZD5991, as it and other MCL1 inhibitors are already undergoing human trials for hematogenous malignancies, with numerous other drugs under development in pre-clinical studies [32]. Canine clinical trials may be an appealing first step in testing MCL1 inhibition in osteosarcoma because AZD5991 and other MCL1 targeting drugs bind to canine MCL1 and human MCL1 with similar affinity, likely due to the fact that MCL1 is nearly identical in these species [39–41]. Dogs are a well-established model of spontaneous osteosarcoma, and the canine disease shares critical genetic, histologic, and pathologic features with osteosarcoma found in people. The only major differences are that osteosarcoma is approximately 10 times more common in dogs than in people, and time to endpoint in canine clinical trials is much shorter than in people [42–49]. In addition to providing the unique opportunity of potentially helping two species at once, this allows for much more rapid testing of potential therapeutics in dogs. By screening promising therapeutics in canine patients first, it may be possible to select therapeutics that have the highest chance of success before moving to human clinical trials.

## Electronic supplementary material

Below is the link to the electronic supplementary material.

Supplementary Material 1

## Data Availability

All scRNAseq data are publicly available in NCBI’s GEO database (GSE234614). Cell lines utilized are available from commercial resources or can be obtained upon request to corresponding author(s); model-specific source information is detailed in the methods section. Imaging datasets will be made available upon request.

## Code Availability

All of the processed data, scripts, and code used to produce the analyses associated with this manuscript are available on GitHub at https://github.com/kidcancerlab/MCL1_early_met_survival. MATLAB code used for the imaging data analyses described herein are available from the authors cited upon request.
