# Supplementary material for: Host-derived growth factors drive ERK phosphorylation and MCL1 expression to promote osteosarcoma cell survival during metastatic lung colonization
Source: Cell Oncol (Dordr). 2023 Sep 7;47(1):259–82. doi: 10.1007/s13402-023-00867-w (PMC10899530; doi:10.1007/s13402-023-00867-w)
Supplement: Supplementary file 1 — Supplementary Material 1 [file 13402_2023_867_MOESM1_ESM.pdf]

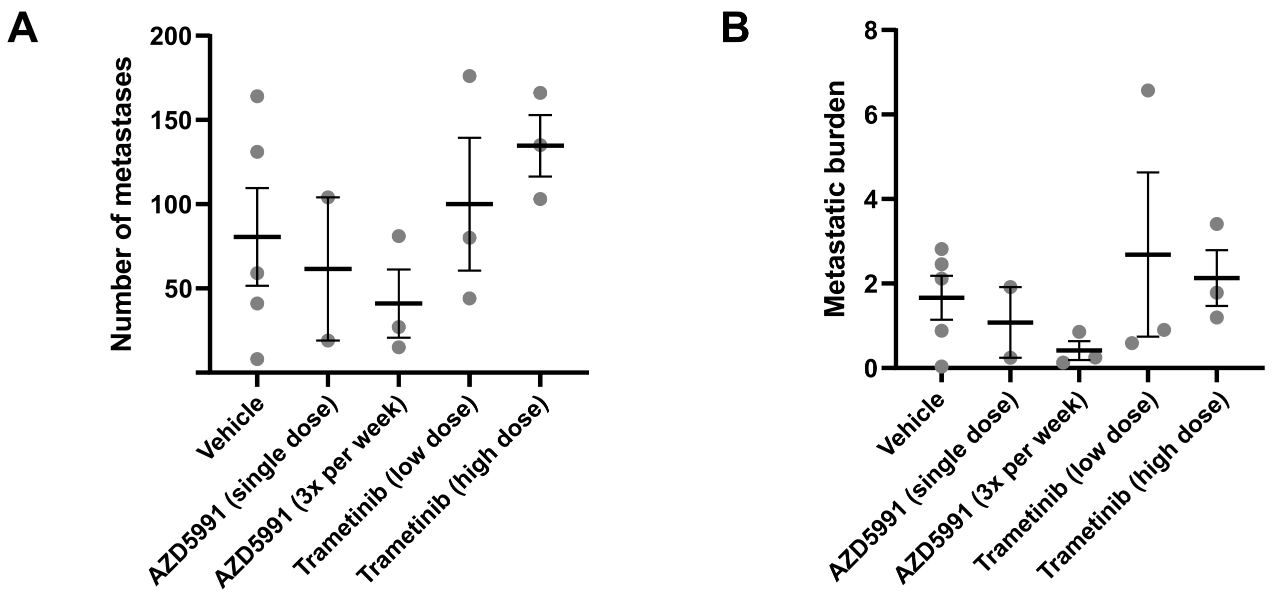

Supplemental Figure 1. A) Dotplot showing the number of metastases counted in each treatment group. Mice that met endpoint prior to end of study were censored from the analysis. A dose-dependent decrease in the mean number of metastases was seen in mice receiving AZD5991 compared to controls. Mice receiving trametinib did not show a similar pattern. B) Dotplot showing metastatic burden (percentage of lung area taken up by tumor in each treatment group. Mice that met endpoint prior to end of study were censored from the analysis. A dose-dependent decrease in the mean metastatic burden was seen in mice receiving AZD5991 compared to controls. Mice receiving trametinib did not show a similar pattern.

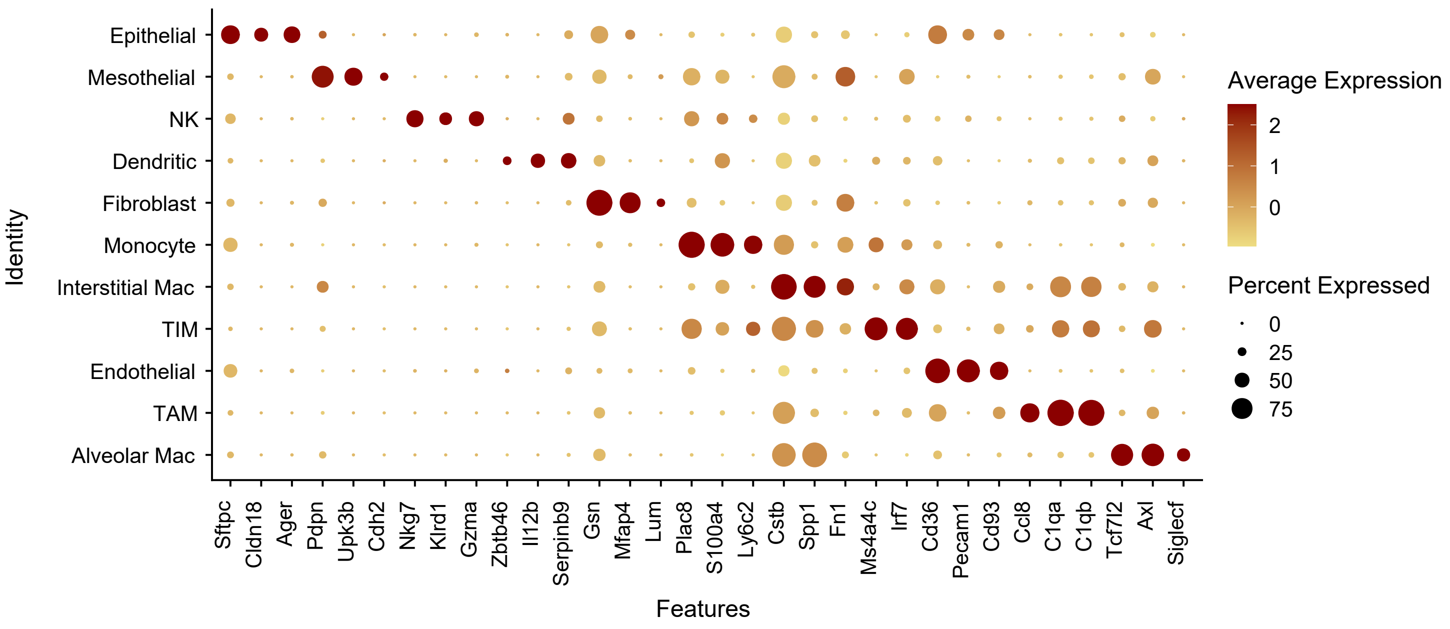

Supplemental Figure 2. Bubble plot in the supplement identifying marker genes validating our cell type assignments for the stromal cells

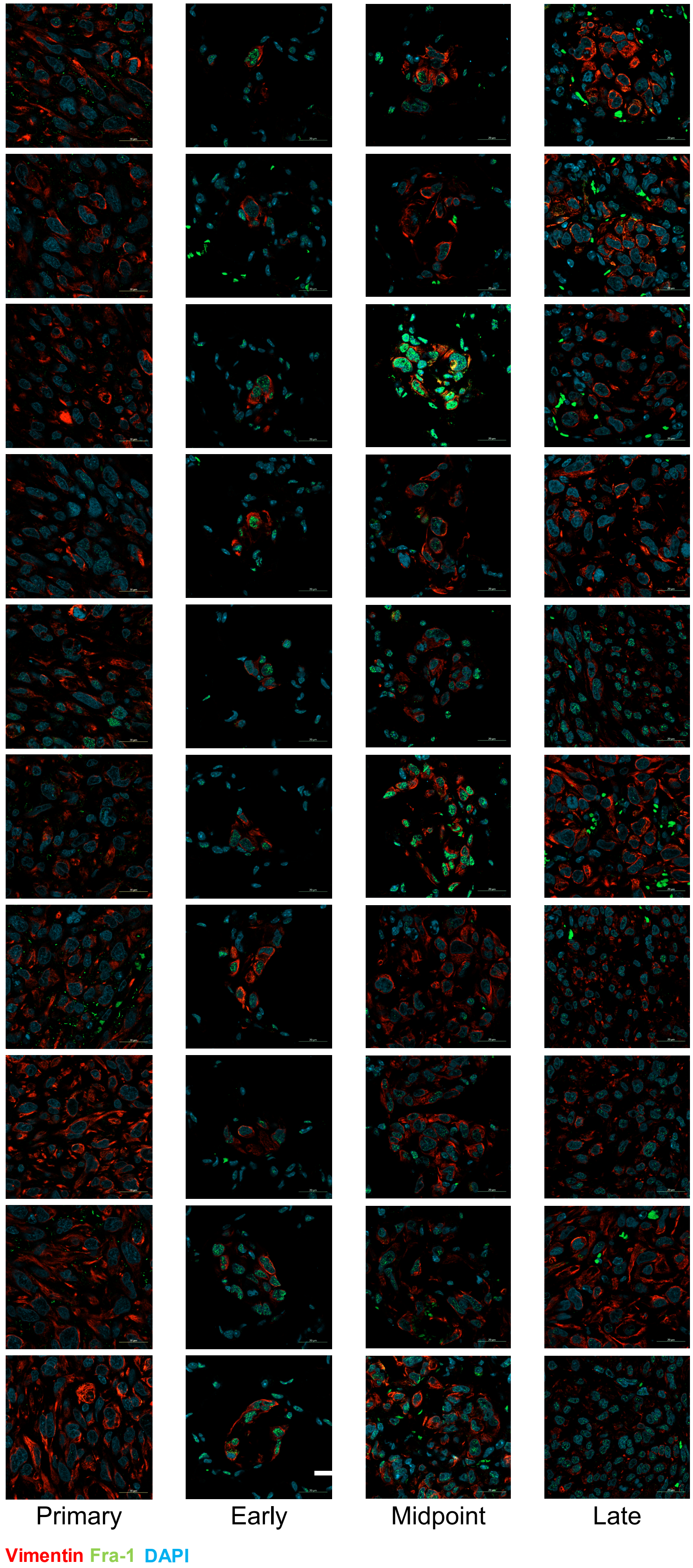

Supplemental Figure 3. Representative images of OS-17 (vimentin, red) captured at each disease timepoint stained for Fra-1 (green). Images are arranged, when possible, from smallest to largest lesion size. DAPI is counterstained in blue. Overall, early metastases express more Fra-1 than primary tumor and later metastases. The image directly to the right of the early timepoint images column is a magnified version of the early timepoint image beside it. Scalebar, 20 µm

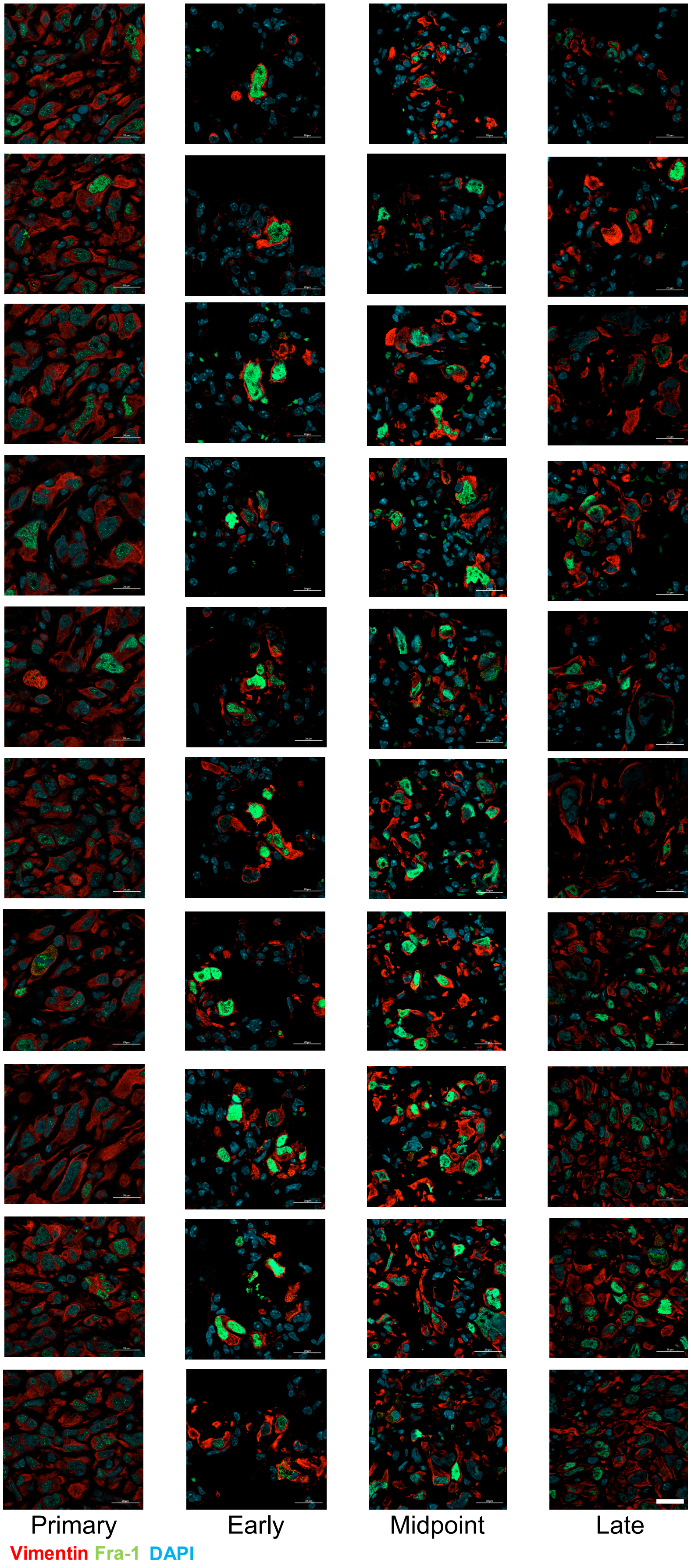

Supplemental Figure 4. Representative images of MG63.3 (vimentin, red) captured at each disease timepoint stained for Fra-1 (green). Images are arranged, when possible, from smallest to largest lesion size. DAPI is counterstained in blue. Overall, a greater proportion of cells within early metastases strongly express Fra-1. Midpoint metastases contain a smaller proportion of strongly Fra-1 positive cells, while primary tumor and late metastases express very little Fra-1.Scalebar, 20 μm

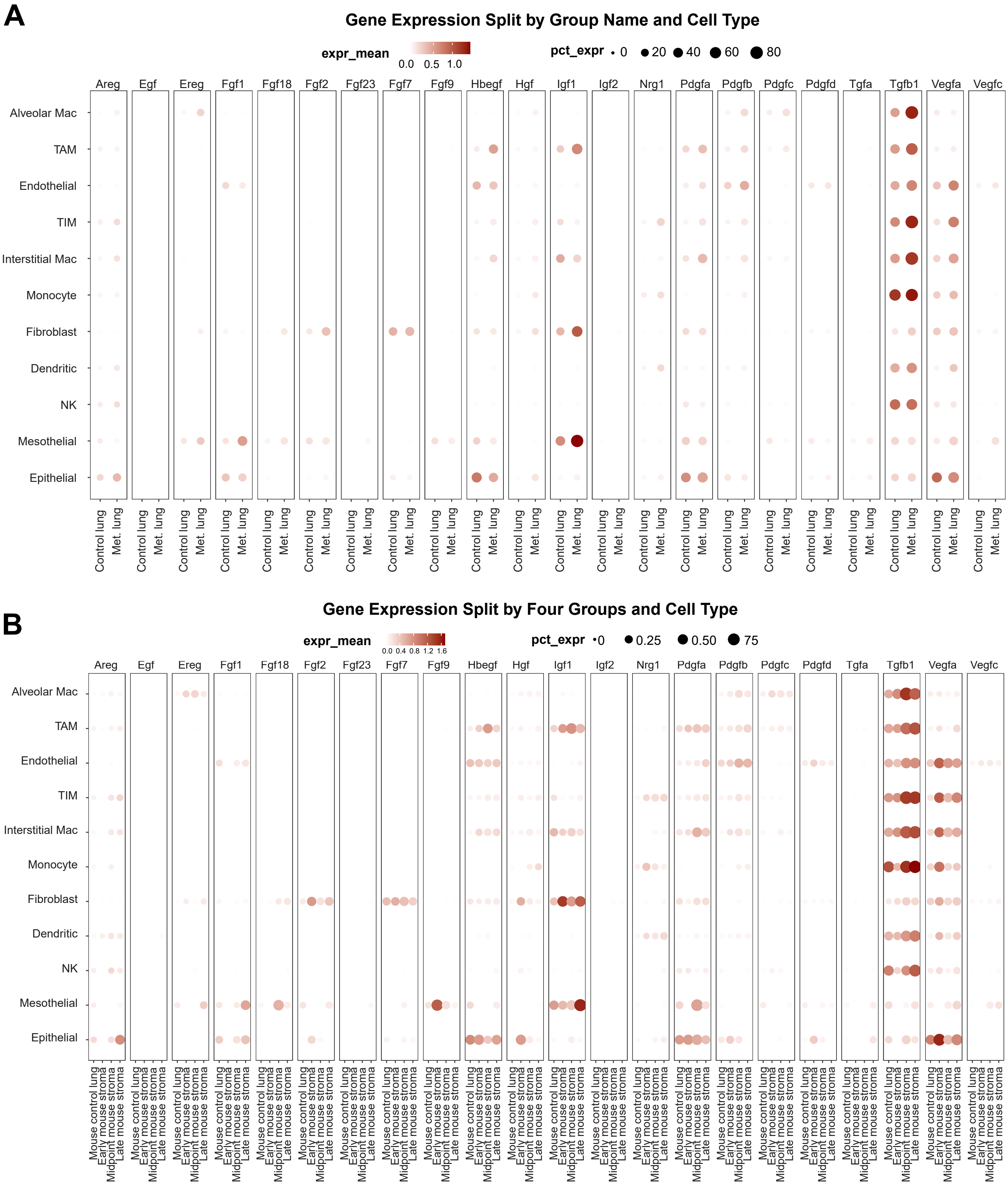

Supplemental Figure 5. A) Dotplot showing growth factor transcription by niche cells in control lungs, and in all metastasis bearing lungs combined. **B)** Dotplot showing growth factor transcription by niche cells in control lungs, and in metastasis-bearing lungs at early, midpoint, and late disease timepoints. A larger percentage of stromal cells transcribe greater amounts of growth factors in metastasis bearing lungs compared to controls.

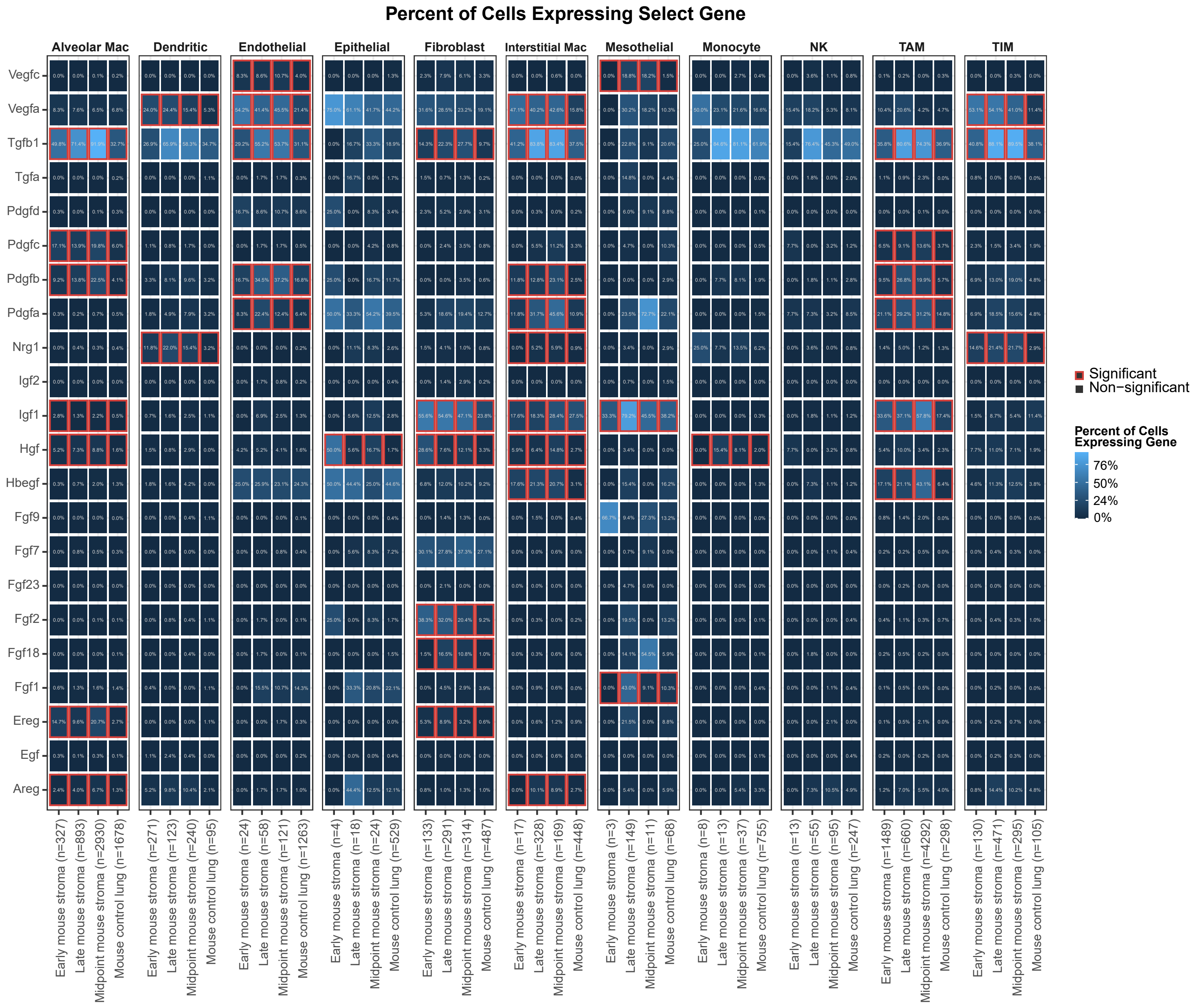

Supplemental Figure 6. Chart showing the percentage of each cell type transcribing the listed growth factor. The percentage of cells transcribing growth factors in tumor-bearing lungs is often higher than the percentage in the control lungs, indicating that growth factor expression is not increased solely due to changes in cell numbers. Significance was determined by Wilcoxon rank sum test. The number of each cell type captured in each population is also shown

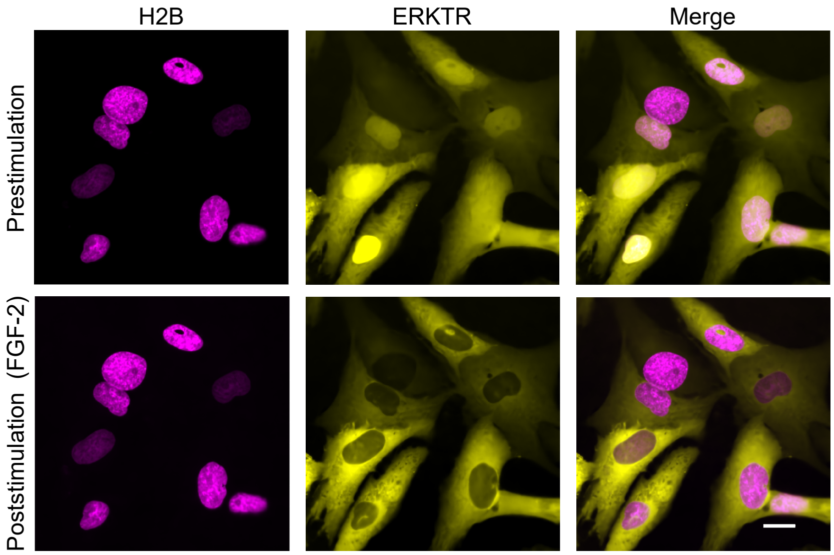

Supplemental Figure 7. Representative images of OS-17 pERK reporter cells before and after stimulation with FGF2. Cellular fluorescence shifts from primarily nuclear to predominantly cytoplasmic, indicating increased ERK phosphorylation. H2B is endogenously expressed, used to identify nuclei. Scalebar, 20 μm.

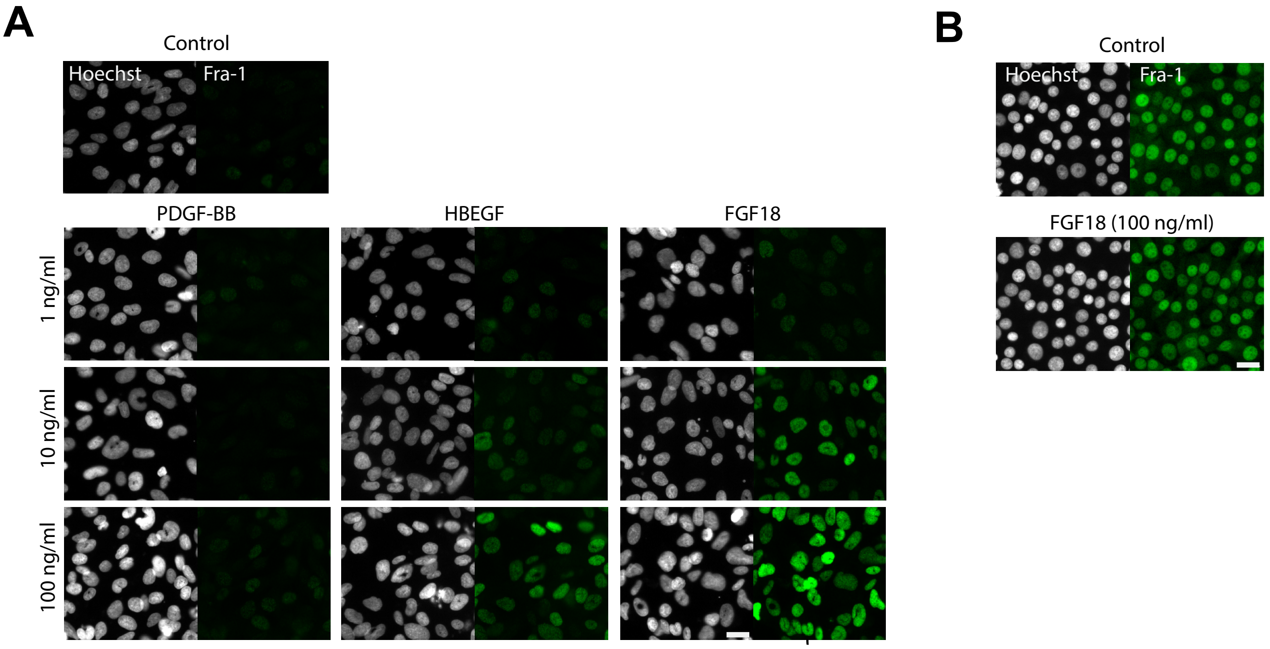

Supplemental Figure 8. A) Representative images of OS-17 cells stained for Fra-1 under control, PDGF-BB, HBEGF and FGF18 treatment conditions at 1, 10 and 100 ng/ml. Cells treated with Class 3 ligand FGF18 show high level of cell-to-cell Fra-1 heterogeneity and a high relative level of expression compared to HBEGF (Class 2) and PDGF-BB(Class 1). Scale bar 25 μm**. B)** Representative images of 143B cells stained for Fra-1 under controland FGF18 treatment conditions for comparison.143B showed near uniform levels of Fra-1 expression Scale bar 25 μm**.**

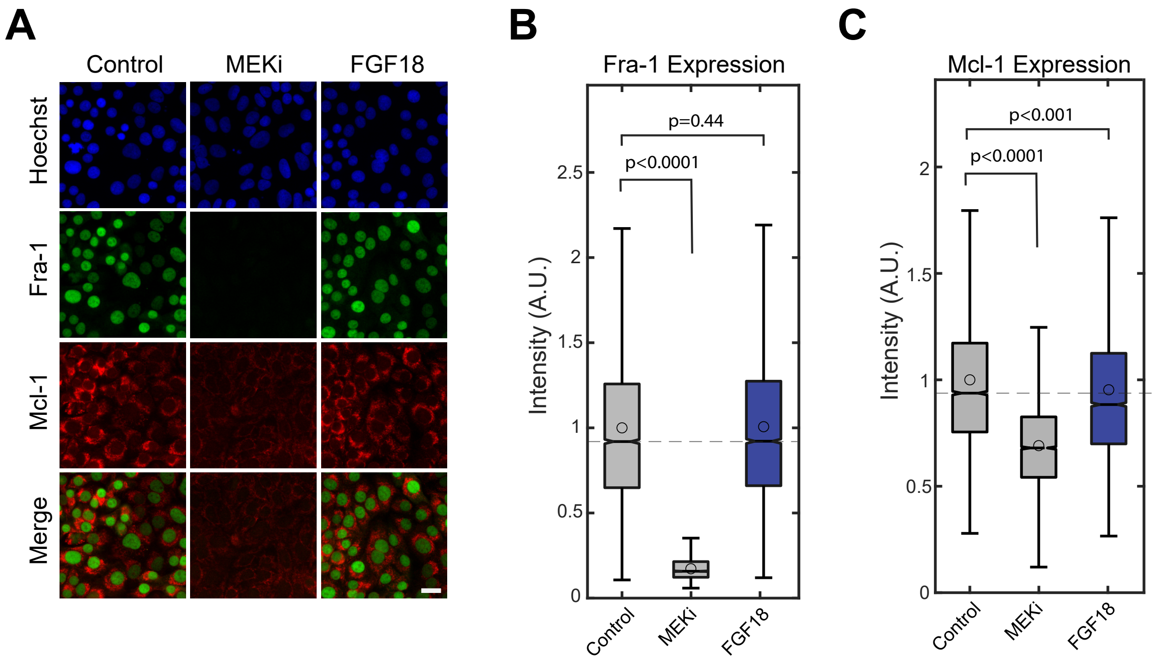

Supplemental Figure 9. A) Representative images of 143B cells stained for Fra-1 and MCL1 under control, MEK inhibitor and FGF18 treatment conditions. Scale bar 25 µm**. B)** Fluorescence intensity of Fra-1 staining in 143B cells exposed FGF18 +/- MEKi. Treatment with MEKi nearly eliminated Fra-1 expression. Treatment with FGF18 has no effect on Fra-1 staining. Dashed line indicates median of control treated cells, circles indicate mean intensity. **C)** Fluorescence intensity of MCL1 staining in 143B cells exposed to FGF18 +/- MEKi. Treatment with MEKi noticeably reduces MCL1 expression. Treatment with FGF18 mildly decreases MCL1 expression compared to control, likely due to pERK saturation at baseline levels and resulting activity of negative feedback loop(s) when cells are exposed to additional growth factors. Dashed line indicates median of control treated cells, circles indicate mean intensity.

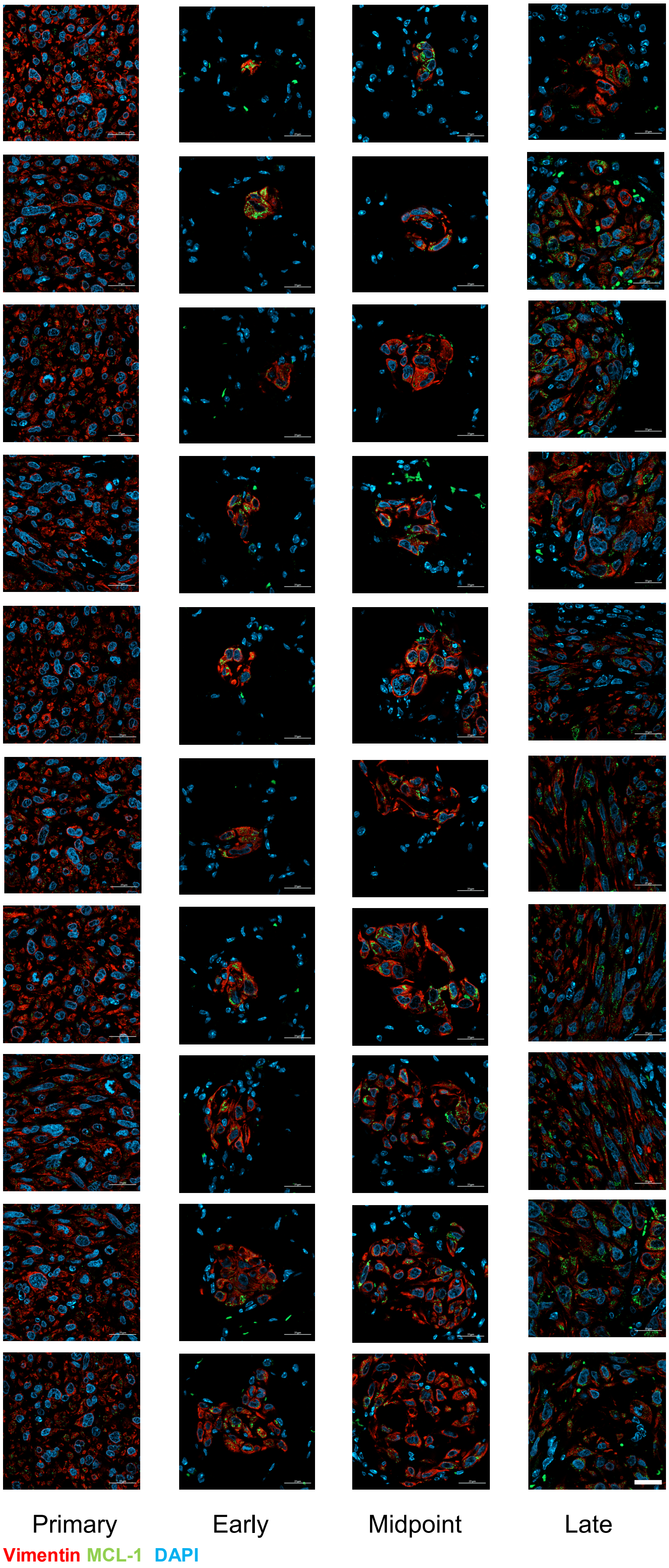

Supplemental Figure 10. Representative images of OS-17 (vimentin, red) captured at each disease timepoint stained for MCL1 (green). Images are arranged, when possible, from smallest to largest lesion size. DAPI is counterstained in blue. Early metastases express more MCL1 than primary tumor and later metastases. Within time points smaller tumors overall express more MCL1 that larger ones.Scalebar, 20 µm

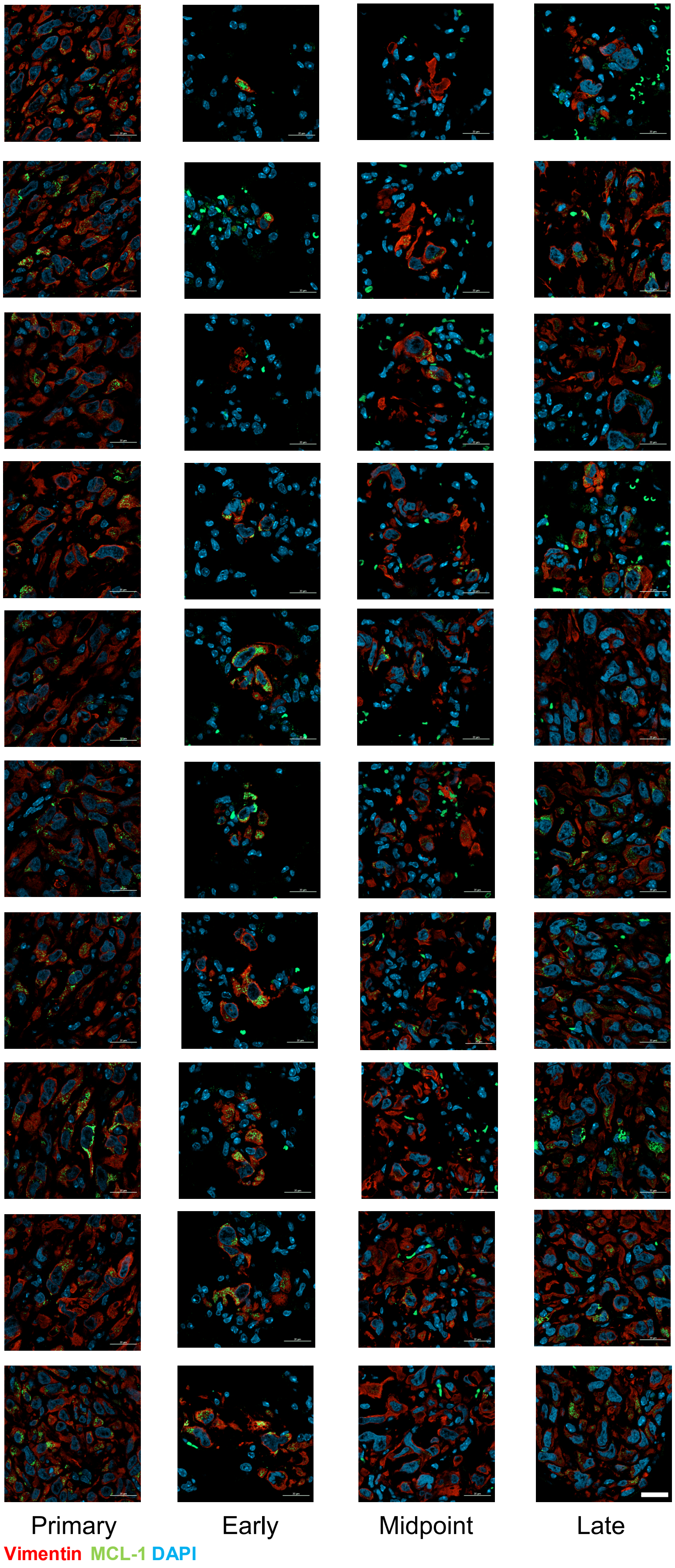

Supplemental Figure 11. Representative images of MG63.3 (vimentin, red) captured at each disease timepoint stained for MCL1 (green). Images are arranged, when possible, from smallest to largest lesion size. DAPI is counterstained in blue. Early metastases express more MCL1 than primary tumor and later metastases. Within time points smaller tumors overall express more MCL1 that larger ones. Scalebar, 20 μm

1. Establish epithelial monolayer

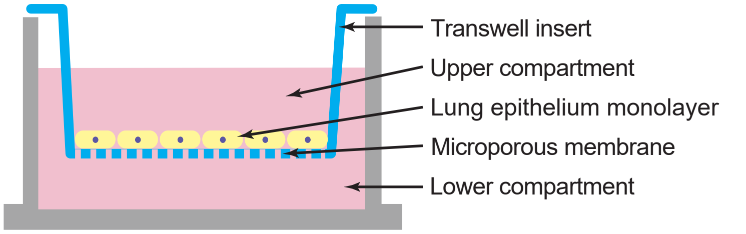

2. Seed tumor cells, establish organoids

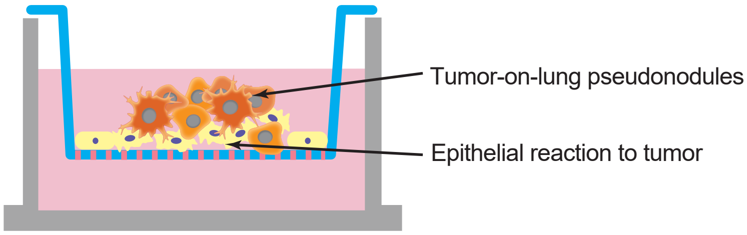

3. Evaluate effect of experimental agent

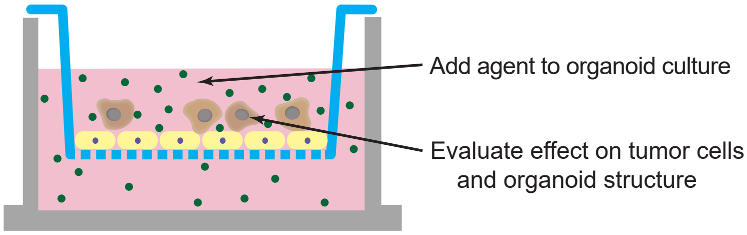

Supplemental Figure 12. Schematic showing creation and evaluation of metastatic organoids. Lung epithelial cells are plated on a microporous membrane and allowed to grow to a confluent monolayer. Tumor cells are added and spontaneously form organoids over 1-3 days. Treatments are added to lower chamber, and diffuse across microporous membrane. This system allows for in vitro modeling of tumor-lung interactions and evaluation of tumor response to treatments in this environment.
